# Therapeutic targeting miR130b counteracts diffuse large B-cell lymphoma progression via OX40/OX40L-mediated interaction with Th17 cells

**DOI:** 10.1038/s41392-022-00895-2

**Published:** 2022-03-18

**Authors:** Rui Sun, Pei-Pei Zhang, Xiang-Qin Weng, Xiao-Dong Gao, Chuan-Xin Huang, Li Wang, Xiao-Xia Hu, Peng-Peng Xu, Lin Cheng, Lu Jiang, Di Fu, Bin Qu, Yan Zhao, Yan Feng, Hong-Jing Dou, Zhong Zheng, Wei-Li Zhao

**Affiliations:** 1 Shanghai Institute of Hematology, State Key Laboratory of Medical Genomics, National Research Center for Translational Medicine at Shanghai, Ruijin Hospital, Shanghai Jiao Tong University of Medicine, Shanghai, China; 2 State Key Laboratory of Metal Matrix Composites, School of Materials Science and Engineering, National Research Center for Translational Medicine at Shanghai, Shanghai Jiao Tong University, Shanghai, China; 3 Department of Immunobiology and Microbiology, Shanghai Institute of Immunology, Shanghai Jiao Tong University School of Medicine, Shanghai, China; 4 Department of Laboratory Medicine, Shanghai RuiJin Hospital, Shanghai Jiao Tong University School of Medicine, Shanghai, China; 5 State Key Laboratory of Microbial Metabolism, School of Life Sciences and Biotechnology, Shanghai Jiao Tong University, Shanghai, China

**Keywords:** Cancer microenvironment, Molecular medicine

## Abstract

MicroRNAs (miRNAs) are involved in lymphoma progression by regulating the tumor microenvironment. Serum miR130b is overexpressed in diffuse large B-cell lymphoma (DLBCL), inducing Th17 cell alterations. To further illustrate its biological significance and therapeutic rationale, miR130b was detected by quantitative real-time PCR in the serum samples of 532 newly diagnosed DLBCL patients. The mechanism of miR130b on lymphoma progression and the tumor microenvironment was investigated both in vitro and in vivo. Therapeutic targeting miR130b was also evaluated, including OX40 agonistic antibody and lipid nanoparticles (LNPs)-miR130b antagomir. The results showed that serum miR130b significantly correlated with tumor miR130b and serum interleukin-17, indicating lymphoma relapse and inferior survival of DLBCL patients. MiR130b overexpression altered tumor microenvironment signaling pathways and increased Th17 cell activity. As mechanism of action, miR130b downregulated tumor OX40L expression by directly targeting IFNAR1/p-STAT1 axis, recruiting Th17 cells via OX40/OX40L interaction, thereby promoting immunosuppressive function of Th17 cells. In co-culture systems of B-lymphoma cells with immune cells, miR130b inhibited lymphoma cell autophagy, which could be counteracted by OX40 agonistic antibody and LNPs-miR130b antagomir. In murine xenograft model established with subcutaneous injection of A20 cells, both OX40 agonistic antibody and LNPs-miR130b antagomir remarkably inhibited Th17 cells and retarded miR130b-overexpressing tumor growth. In conclusion, as an oncogenic biomarker of DLBCL, miR130b was related to lymphoma progression through modulating OX40/OX40L-mediated lymphoma cell interaction with Th17 cells, attributing to B-cell lymphoma sensitivity towards OX40 agonistic antibody. Targeting miR130b using LNPs-miR130b antagomir could also be a potential immunotherapeutic strategy in treating OX40-altered lymphoid malignancies.

## Introduction

Diffuse large B-cell lymphoma (DLBCL) is the most prevalent subtype of aggressive B-cell lymphoma. Although significant progress has been achieved on anti-CD20 antibody rituximab-containing immunochemotherapy, the clinical outcome of relapsed or refractory patients is still dismal, with a median survival time shorter than 6 months.^1^ The morbidity of DLBCL is 1.8 per 100,000 persons per year according to the recent statistical data from NCI (Surveillance, Epidemiology, and End Results, SEER Program).^2^ In addition to the genetic abnormalities of lymphoma cells themselves, microenvironmental immune cells dysfunction can lead to tumor progression.^3^ However, the underlying mechanism of lymphoma cell escape from tumor immunity needs further investigation.

Immune checkpoint modulators have emerged as successful targeted therapeutic agents for multiple cancers, including lymphoma. OX40 and its ligand OX40L are members of the tumor necrosis factor superfamily, playing a pivotal role in promoting T-cell survival and regulating cytokine receptor signaling.^4^ OX40 is expressed predominantly on T cells, while OX40L is expressed on activated antigen-presenting cells including B cells, dendritic cells, and macrophages.^5^ Monoclonal agonistic antibodies that activate OX40 have been used to augment immune responses against tumor cells.^6^ Clinically, humanized OX40 agonistic antibody is effective and well tolerated in patients with advanced solid tumors.^7^ By targeting immune cells, OX40/OX40L interaction can suppress Th17 cell persistence and the secretion of its main cytokine interleukin-17 (IL17).^8^ Although the role of Th17 cells in autoimmune disorders and inflammation has been studied, it is still controversial how Th17 cells affect tumor immunity. On one hand, Th17 cells may induce tumor growth by influencing immunosuppressive functions. On the other hand, Th17 cells could modulate antitumor immune responses by recruiting immune cells into tumors, converting toward Th1 phenotype, or even activating CD8 T cells and producing IFN-γ.^9^ Recent data on DLBCL showed that aberrant Th17 cells were significantly associated with inferior survival of the patients.^10^ Therefore, therapeutic targeting OX40/OX40L interaction with downstream Th17 cells remains of great interest for exploring potential immunotherapeutic approaches in DLBCL.

MicroRNAs (miRNAs) are 19–24 nucleotides long non-coding RNA that regulate gene expression by targeting the 3′-untranslated region of mRNA. Antagomirs are chemically complete sequences of the corresponding miRNAs that block miRNA function.^11^ Serum miRNAs are non-invasive biomarkers and can be therapeutically targeted by miRNA antagomirs.^12^ Lipid nanoparticles (LNPs) have been revealed as promising carriers of miRNA antagomirs, remarkably improving the intracellular endocytosis of antagomirs into cancer cells and efficiently inhibiting tumor growth.^13^ Recently, we proposed a prognostic model of four circulating miRNA (miR21, miR130b, miR155, and miR28), which contributes to DLBCL progression through altering myeloid-derived suppressor cells (MDSCs) and Th17 cells.^14^ Among these miRNAs, miR21 and miR155 are well-known oncogenic biomarkers and have been shown to provoke cancer progression by acting on immunosuppressive MDSCs and Th17 cells.^15–18^ In the present study, we further investigated the biological significance and therapeutic rationale of miR130b on the tumor microenvironment in DLBCL both in vitro and in vivo.

## Results

### Elevated serum miR130b was significantly associated with lymphoma progression in DLBCL

**Fig. 1:**
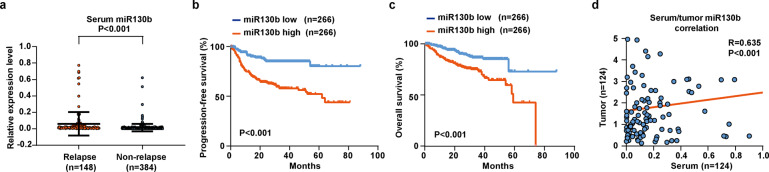
Elevated serum miR130b was significantly associated with lymphoma progression in DLBCL. **a** Real-time PCR analysis of miR130b expression in relapse patients (*n* = 148) and non-relapse patients (n = 384). **b**, **c** Progression-free survival (PFS) (**b**) and overall survival (OS) curves (**c**) in DLBCL patients according to miR130b expression. **d** Correlation between serum miR130b and tumor miR130b expression (*n* = 124) calculated by Pearson correlation coefficient analysis

To confirm the clinical significance of miR130b in DLBCL, we first assessed expression of serum miR130b in a large cohort of 532 newly diagnosed patients, including 148 relapse (referred to disease relapse after achieving remission^19^) and 384 non-relapse patients. The median expression value of miR130b was 0.129 in DLBCL. The miR130b high group referred to patients with a miR130b expression level over or equal to the median value, whereas the miR130b low group included those below the median value. Compared with non-relapse patients, serum miR130b was significantly elevated in relapse patients (*P* < 0.001, Fig. 1a). The relapse rate was 41.7% for miR130b high group, significantly higher than that of miR130b low group (13.9%, *P* < 0.001). With a median follow-up time of 51.6 months (range 0.2–88.0 months), the 2-year progression-free survival (PFS) and overall survival (OS) of the patients were 73.4% and 83.1%, respectively. Univariate analysis (Supplementary Table 1) and multivariate analysis (Supplementary Table 2) were performed for predictors of PFS and OS. The 2-year PFS was 63.0% for miR130b high group and 84.3% for miR130b low group (*P* < 0.001, Fig. 1b). The 2-year OS was 76.7% for miR130b high group and 89.9% for miR130b low group (*P* < 0.001, Fig. 1c). By multivariate analysis, miR130b expression was an independent prognostic factor for inferior PFS and OS when IPI was controlled (*P* < 0.001 and *P* = 0.008). Serum and tumor miR130b expression was significantly correlated using Pearson correlation coefficient analysis (*R* = 0.635, *P* < 0.001, Fig. 1d). These data revealed that miR130b contributed to DLBCL progression.

### MiR130b affected signaling pathways involved in the tumor microenvironment of DLBCL

**Fig. 2:**
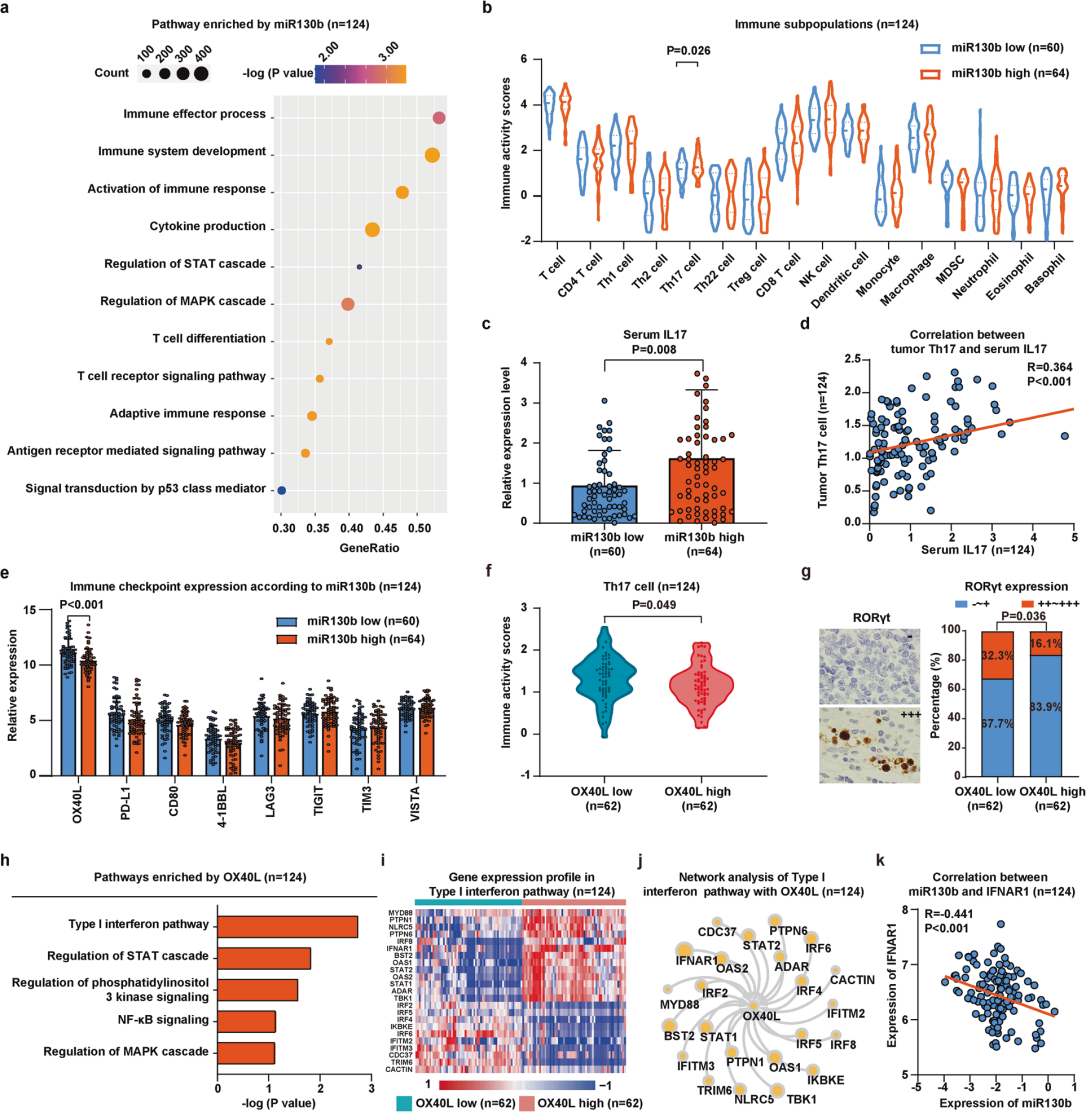
MiR130b affected signaling pathways involved in the tumor microenvironment of DLBCL. **a** Differentially expressed gene ontology (GO) terms according to miR130b expression. −Log (*P* value) of dysregulated pathways was indicated by color of points using RNA-sequencing. The size of points indicates the number of genes included in each gene set. **b** Distribution of immune subpopulations in miR130b high group (*n* = 64) and miR130b low group (*n* = 60) analyzed by transcriptomics data using TIP analysis. **c** Serum IL17 level of DLBCL patients measured by ELISA in miR130b high group (*n* = 64) and miR130b low group (*n* = 60). Data are summarized as mean ± SD. **d** Correlation between serum IL17 and tumor Th17 cell (*n* = 124) calculated by Pearson correlation coefficient analysis. **e** Differentially expressed immune checkpoints identified by RNA-sequencing between miR130b high group (*n* = 64) and miR130b low group (*n* = 60). **f** The immune activity scores of Th17 cells of DLBCL patients in OX40L low group (*n* = 62) and OX40L high group (*n* = 62). **g** Immunohistochemical study of Th17 cells in tumor samples of DLBCL patients in OX40L low group (*n* = 62) and OX40L high group (*n* = 62). **h** Pathway enrichment analysis in DLBCL patients according to OX40L expression using gene ontology database. **i** Heatmap diagram of the differentially expressed gene in type I interferon pathway between OX40L low group (*n* = 62) and OX40L high group (*n* = 62). Mean expression values are shown. Red, increased expression; blue, decreased expression; and white, mean value. **j** RNA-sequencing was used to determine gene–gene interaction network of type I interferon pathway. Genes that were more likely to be functionally related were presented as larger nodes. **k** Correlation between miR130b and IFNAR1 expression (*n* = 124) calculated by Pearson correlation coefficient analysis

To explore the biological significance of miR130b, we then performed RNA-sequencing analysis on tumor samples of 124 patients. Gene Set Enrichment Analysis (GSEA) manifested that immune-associated signaling pathways (immune effector process, immune system development, activation of immune response, cytokine production, T cell differentiation, T cell receptor signaling pathway, adaptive immune response, antigen receptor-mediated signaling pathway), as well as oncogenic signaling pathways (regulation of STAT cascade, regulation of MAPK cascade and signal transduction by p53 class mediator), were differentially expressed according to miR130b expression (Fig. 2a). Meanwhile, the immune activity scores of immune cells were calculated by TIP analysis, including T-cell subsets, monocyte, macrophage, MDSC, dendritic cell, neutrophil, and natural killer cell. Among them, Th17 cells were significantly higher in miR130b high group than in miR130b low group (*P* = 0.026, Fig. 2b). Acting as the main cytokine secreted by Th17 cells,^20^ serum IL17 level was accordingly increased in miR130b high group, compared to miR130b low group (*P* = 0.008, Fig. 2c). Tumor Th17 cells manifested a significant correlation with serum IL17 level (*R* = 0.364, *P* < 0.001, Fig. 2d).

To further determine the potential mechanism of miR130b on Th17 cells, we also analyzed tumor cell expression of immune checkpoint genes. OX40L was the most differentially expressed immune checkpoint gene between miR130b high group and miR130b low group (*P* < 0.001, Fig. 2e). TIP analysis also revealed that Th17 cells were increased significantly in OX40L low group than in OX40L high group (*P* = 0.049, Fig. 2f). As revealed in tumor samples of DLBCL patients by immunohistochemistry, RORγt-positive Th17 cells were more frequently detected in OX40L low group than in OX40L high group (*P* = 0.036, Fig. 2g).

According to OX40L expression, multiple signaling pathways enriched in the OX40L high group included type I interferon pathway, regulation of STAT cascade, regulation of phosphatidylinositol 3 kinase signaling, NF-κB signaling, and regulation of MAPK cascade (Fig. 2h). The 23 differentially expressed genes of the type I interferon pathway were plotted by heatmap in Fig. 2i, including IFNAR1, STAT1, BST2, OAS1, TBK1, IRF6, OAS2, STAT2, PTPN6, NLRC5, IRF4, ADAR, IKBKE, PTPN1, IRF2, IRF5, TRIM6, IFITM3, CDC37, IFITM2, IRF8, CACTIN, and MYD88. The interaction of the above 23 genes with OX40L were visualized by cytoscape program, with IFNAR1 presenting the largest node and identifying as the most functionally related gene (Fig. 2j). Notably, miR130b and IFNAR1 displayed significant correlation by the Pearson correlation coefficient analysis (*R* = −0.441, *P* < 0.001, Fig. 2k).

Together, miR130b expression was significantly associated with Th17 cell accumulation in DLBCL, probably through IFNAR1 and OX40L modulation.

### MiR130b modulated OX40/OX40L-mediated B-lymphoma cell interaction with Th17 cells via IFNAR1/p-STAT1 axis

To verify the effects of miR130b on IFNAR1 expression, miR130b mimics and miR130b inhibitor were utilized to regulate miR130b in DB cells and OCI-ly10 cells. According to miR130b expression (*P* < 0.001, Supplementary Fig. 1a), DB cells with lower miR130b expression were transfected with miR130b mimics, and OCI-ly10 cells with higher miR130b expression were transfected with miR130b inhibitor. Comparing with the control mimics, DB cells transfected with miR130b mimics resulted in significantly increased miR130b expression (*P* < 0.001, Supplementary Fig. 1b). Comparing with the control inhibitor, OCI-ly10 cells transfected with miR130b inhibitor resulted in significantly decreased miR130b expression (*P* = 0.003, Supplementary Fig. 1b).

The gene expression of the key factors and marker genes of type I interferon pathway were assessed by quantitative real-time PCR, including IFNAR1, IFNAR2, STAT1, STAT2, STAT3, and OX40L. IFNAR1 and OX40L expression were downregulated on DB cells transfected with miR130b mimics (*P* < 0.001 and *P* = 0.047, Supplementary Fig. 1c), and upregulated on OCI-ly10 cells transfected with miR130b inhibitor (*P* < 0.001 and *P* = 0.019, Supplementary Fig. 1c). No significant difference was observed on IFNAR2, STAT1, STAT2, and STAT3 expression (Supplementary Fig. 1c). As assessed by western blot, IFNAR1, p-STAT1, and OX40L expression were downregulated on DB cells transfected with miR130b mimics, and upregulated on OCI-ly10 cells transfected with miR130b inhibitor (Supplementary Fig. 1d). These data suggested that miR130b promoted the crosstalk between B-lymphoma cells and Th17 cells of the tumor microenvironment by IFNAR1 and OX40L.

**Fig. 3:**
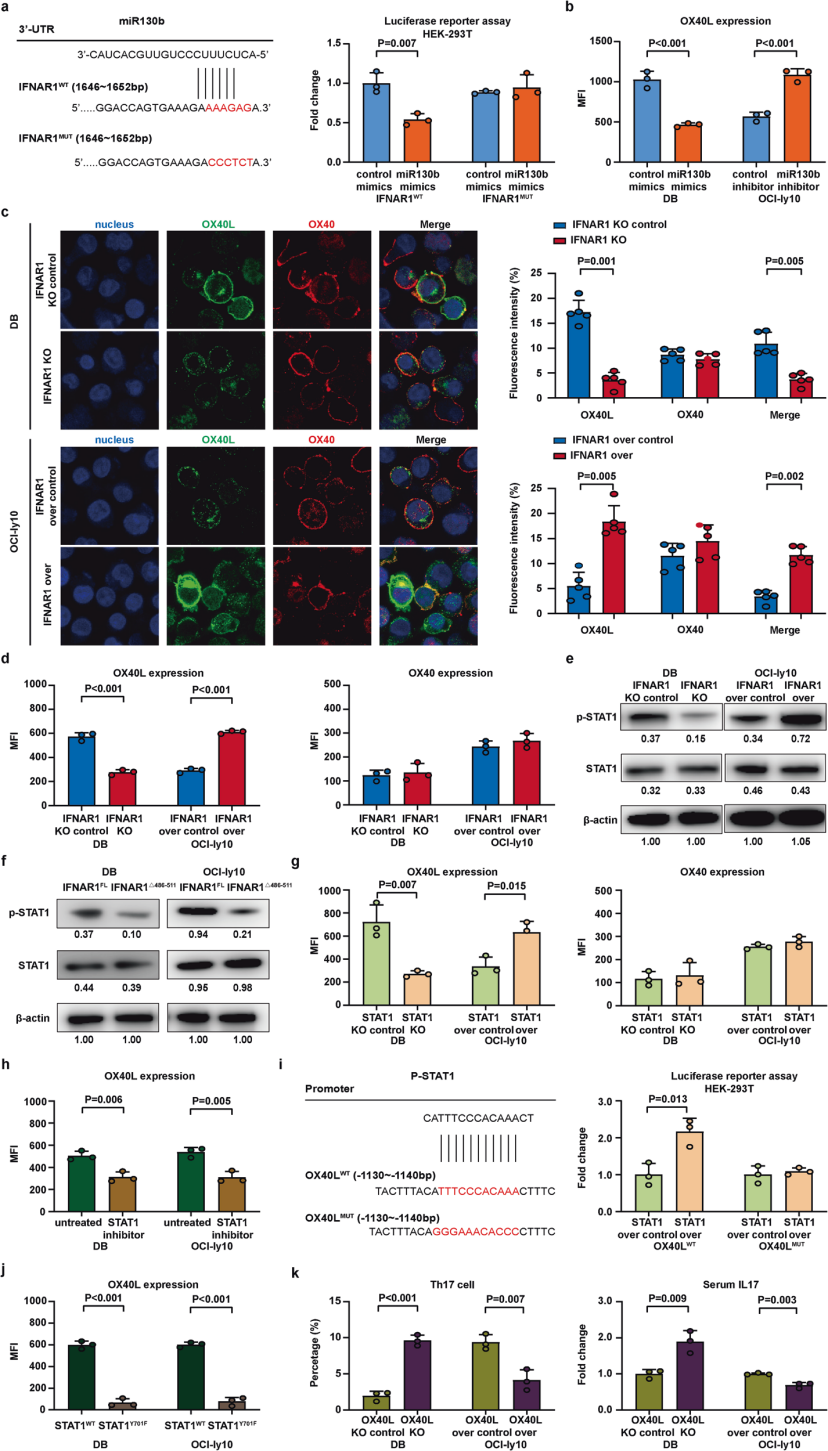
MiR130b modulated OX40/OX40L-mediated B-lymphoma cell interaction with Th17 cells via IFNAR1/p-STAT1 axis. **a** Potential binding sites of miR130b with the 3′-UTR of IFNAR1 (left panel) were predicted by bioinformatics analysis and the relative luciferase activities in HEK-293T cells transfected with IFNAR1^WT^ or IFNAR1^MUT^ were assessed. Data are summarized as mean ± SD (*n* = 3). **b** Flow cytometry analysis of OX40L expression on lymphoma cells in the miR130b-overexpressing DB co-culture system and miR130b-knockdown OCI-ly10 co-culture system. **c** Immunofluorescence assay of OX40 and OX40L in the IFNAR1-knockdown DB co-culture system and IFNAR1-overexpressing OCI-ly10 co-culture with Th17 cells. Representative immunofluorescent visions of OX40L [green]/OX40 [red] and nucleus counterstained with DAPI [blue]). The fluorescence intensity of OX40L, OX40, and OX40/OX40L interaction were quantified using the Image J software from five visions selected at random and subjected for statistical analysis. Data are summarized as mean ± SD (*n* = 5). **d** Flow cytometry analysis of OX40L expression on lymphoma cells and OX40 expression on Th17 cells in the IFNAR1-knockdown DB co-culture system and IFNAR1-overexpressing OCI-ly10 co-culture system. **e** Western blot analysis of p-STAT1 and STAT1 expression in IFNAR1-knockdown DB cells and IFNAR1-overexpressing OCI-ly10 cells. **f** Western blot analysis of p-STAT1 and STAT1 expression in IFNAR1^Δ486-511^ DB cells and IFNAR1^Δ486-511^ OCI-ly10 cells. **g** Flow cytometry analysis of OX40L expression on lymphoma cells and OX40 expression on Th17 cells in the STAT1-knockdown DB co-culture system and STAT1-overexpressing OCI-ly10 co-culture system. Data are summarized as mean ± SD (*n* = 3). **h** Flow cytometry analysis of OX40L expression on lymphoma cells in DB cells and OCI-ly10 cells upon treatment with p-STAT1 inhibitor. Data are summarized as mean ± SD (*n* = 3). **i** Potential binding sites of p-STAT1 with OX40L promotor region (−1130 bp to −1140 bp) predicted by bioinformatics analysis and the relative luciferase activities in HEK-293T cells transfected with OX40L^WT^ or OX40L^MUT^. Data are summarized as mean ± SD (*n* = 3). **j** Flow cytometry analysis of OX40L expression in STAT1^Y701F^ DB cells and STAT1^Y701F^ OCI-ly10 cells. Data are summarized as mean ± SD (*n* = 3). **k** Th17 cell percentage and IL17 level were assessed in the OX40L-knockdown DB co-culture system and OX40L-overexpressing OCI-ly10 co-culture system. Th17 cell percentage (CD4+ RORγt+) was assessed by flow cytometry. IL17 level was measured by ELISA. Data are summarized as mean ± SD (*n* = 3)

To further determine how miR130b exerts its biological function on IFNAR1, we predicted potential binding regions of IFNAR1 3′-UTR (1642–1652 bp) with miR130b using Bioinformatics analysis. As verified via luciferase reporter assay, miR130b negatively modulated the transcriptional activity in IFNAR1^WT^ HEK-293T cells (*P* = 0.007, Fig. 3a), which was not observed in IFNAR1^MUT^ HEK-293T cells (Fig. 3a), indicating that miR130b targeted IFNAR1 through the 3′-UTR binding region. To mimic the in vivo situation, we co-cultured lymphoma cells with peripheral blood mononuclear cells (PBMCs) and lymphoma cells were sorted by EasySep^TM^ Human CD20+ Cell Isolation Kit for further study. We performed flow cytometry to examine the role of miR130b in OX40L on lymphoma cells. OX40L expression was decreased in miR130b mimics transfected DB co-culture system, but increased in miR130b inhibitor transfected OCI-ly10 co-culture system (*P* both <0.001, Fig. 3b and Supplementary Fig. 2a). Moreover, we performed multicolor flow cytometry to examine the role of miR130b in immune checkpoint genes including PD-L1, CD80, 4-1BBL, LAG3, TIGHT, TIM3, and VISTA on lymphoma cells in the miR130b-overexpressing DB co-culture system and miR130b-knockdown OCI-ly10 co-culture system. No significant difference was observed on these immune checkpoint genes (Supplementary Fig. 3a, b). Therefore, we proved that miR130b could regulate OX40/OX40L. As revealed by immunofluorescence assay, OX40/OX40L-mediated B-lymphoma cell crosstalk with Th17 cells was inhibited in the IFNAR1-knockdown DB co-culture system (*P* = 0.001 and *P* = 0.005), but enhanced in the IFNAR1-overexpressing OCI-ly10 co-culture system (*P* = 0.005 and *P* = 0.002, Fig. 3c). Flow cytometry showed that OX40L expression on DB cells was inhibited in the IFNAR1-knockdown DB co-culture system (*P* < 0.001), but enhanced in the IFNAR1-overexpressing OCI-ly10 co-culture system (*P* < 0.001), with OX40 expression remained constant on Th17 cells (Fig. 3d and Supplementary Fig. 2b, c).

We subsequently investigated the role of p-STAT1 in the regulation of IFNAR1 and OX40L expression. p-STAT1 expression was downregulated in IFNAR1-knockdown DB cells, but upregulated in IFNAR1-overexpressing OCI-ly10 cells (Fig. 3e). To futher determine the direct interaction of IFNAR1 with p-STAT1, we predicted the cytoplasmic domain (486aa to 511aa) of IFNAR1 could activate p-STAT1 expression.^21^ Next, we constructed the plasmid with full-length IFNAR1 (IFNAR1^FL^) and with specific deletion of cytoplasmic domain 486aa to 511aa (IFNAR1^Δ486–511^). As assessed by western blot, p-STAT1 expression was decreased in IFNAR1^Δ486–511^ DB cells and IFNAR1^Δ486–511^ OCI-ly10 cells (Fig. 3f), as compared to IFNAR1^FL^ cells (Fig. 3f).

Meanwhile, OX40L expression was downregulated in the STAT1-knockdown DB co-culture system (*P* = 0.007, Fig. 3g), but upregulated in the STAT1-overexpressing OCI-ly10 co-culture system (*P* = 0.015, Fig. 3g), with OX40 expression remained constant on Th17 cells (Fig. 3g and Supplementary Fig. 2d, e). Similarily, OX40L expression was downregulated in DB cells and OCI-ly10 cells upon treatment with p-STAT1 inhibitor (*P* = 0.006 and *P* = 0.005, Fig. 3h and Supplementary Fig. 2f). To further determine the direct interaction of p-STAT1 with OX40L, we predicted the potential binding regions of p-STAT1 with OX40L promoter (−1130 bp to −1140 bp). Next, we constructed the plasmid with OX40L^WT^ and OX40L^MUT^. As detected by luciferase reporter assay, p-STAT1 positively regulated OX40L transcriptional activity in OX40L^WT^ HEK-293T cells, which was not observed in OX40L^MUT^ HEK-293T cells (*P* = 0.013, Fig. 3i). To further verify that phosphorylation of STAT1 tyrosine-701 is critical for upregulation of OX40L expression, we constructed the plasmid with STAT1^WT^ and with point mutation tyrosine-701(STAT1^Y701F^). OX40L expression was decreased in STAT1^Y701F^ DB cells and STAT1^Y701F^ OCI-ly10 cells, as compared to STAT1^WT^ cells (*P* both <0.001, Fig. 3j and Supplementary Fig. 2g).

To illustrate the regulatory role of OX40L on the accumulation of Th17 cells, we established OX40L-knockdown DB cells and OX40L-overexpressing OCI-ly10 cells. Th17 cell percentage and IL17 level were significantly increased in the OX40L-knockdown DB co-culture system (*P* < 0.001 and *P* = 0.009, Fig. 3k), but decreased in the OX40L-overexpressing OCI-ly10 co-culture system (*P* = 0.007 and *P* = 0.003, Fig. 3k and Supplementary Fig. 2h).

Together, miR130b could regulate OX40/OX40L interaction between B-lymphoma cells and Th17 cells through modulating IFNAR1/p-STAT1 axis.

### MiR130b induced Th17 cell accumulation and IL17 secretion

**Fig. 4:**
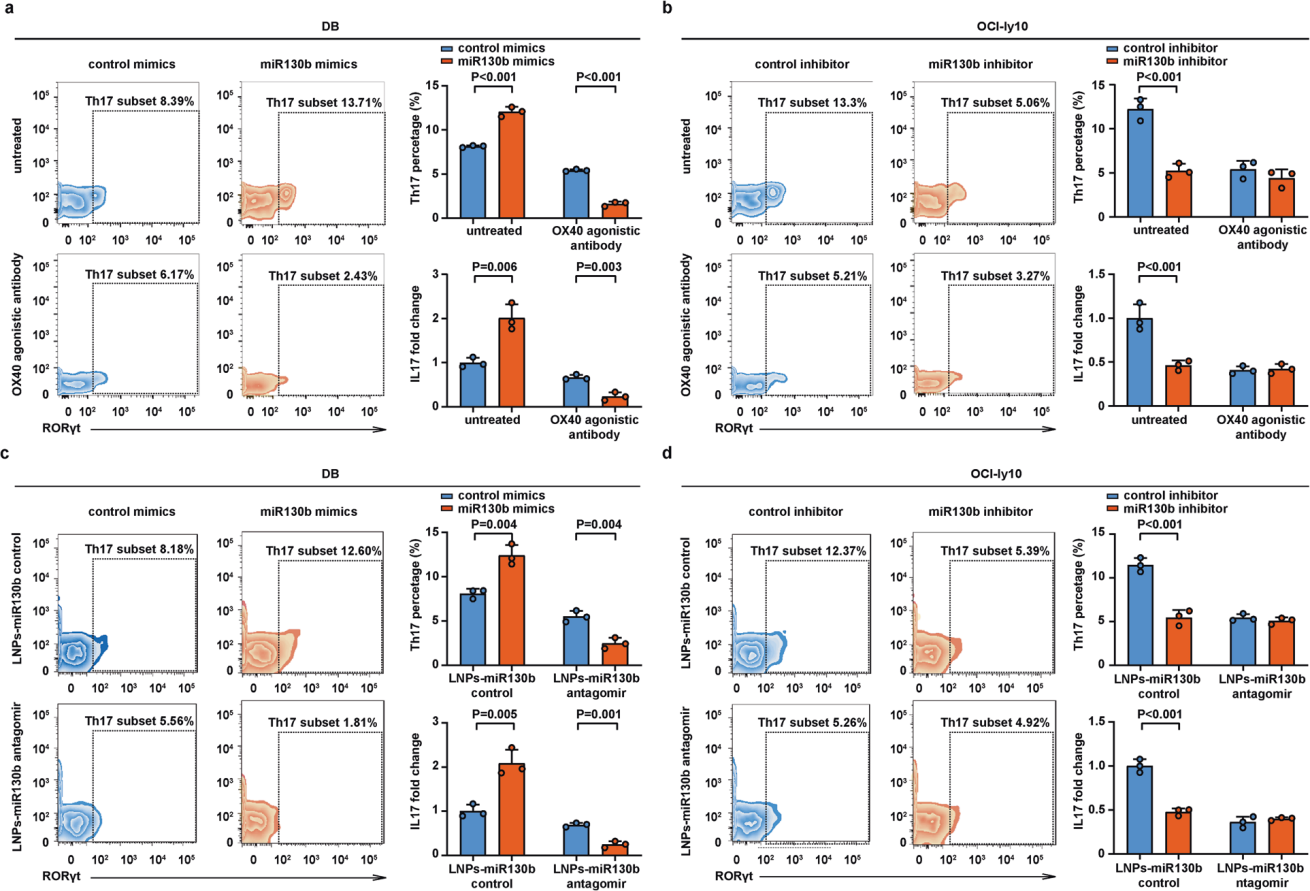
MiR130b induced Th17 cell accumulation and IL17 secretion. **a** Th17 cell percentage and IL17 level were assessed in the control mimics-transfected DB co-culture system and miR130b mimics-transfected DB co-culture system upon treatment with or without OX40 agonistic antibody. Th17 cell percentage (CD4+ RORγt+) was assessed by flow cytometry. IL17 level was measured by ELISA. Data are summarized as mean ± SD (*n* = 3). **b** Th17 cell percentage and IL17 secretion were assessed in the control inhibitor-transfected OCI-ly10 co-culture and miR130b inhibitor-transfected OCI-ly10 co-culture upon treatment with or without OX40 agonistic antibody. Th17 cell percentage (CD4+ RORγt+) was assessed by flow cytometry. IL17 level was measured by ELISA. Data are summarized as mean ± SD (*n* = 3). **c** Th17 cell percentage and IL17 level were assessed in the control mimics-transfected DB co-culture system and miR130b mimics-transfected DB co-culture system upon treatment with or without LNPs-miR130b antagomir. Th17 cell percentage (CD4+ RORγt+) was assessed by flow cytometry. IL17 level was measured by ELISA. Data are summarized as mean ± SD (*n* = 3). **d** Th17 cell percentage and IL17 level were assessed in the control inhibitor-transfected OCI-ly10 co-culture and miR130b inhibitor-transfected OCI-ly10 co-culture upon treatment with or without LNPs-miR130b antagomir. Th17 cell percentage (CD4+ RORγt+) was assessed by flow cytometry. IL17 level was measured by ELISA. Data are summarized as mean ± SD (*n* = 3)

We next examined the role of miR130b in Th17 cell percentage and function in the tumor microenvironment, as well as the therapeutic effect of OX40 agonistic antibody and LNPs-miR130b antagomir. Th17 cell percentage (*P* < 0.001, Fig. 4a) and IL17 level (*P* = 0.006, Fig. 4a) were significantly elevated in the miR130b-overexpressing DB co-culture system, but reduced in the miR130b-overexpressing DB co-culture system upon treatment with OX40 agonistic antibody (*P* < 0.001 and *P* = 0.003, Fig. 4a). Also, Th17 cell percentage (*P* < 0.001, Fig. 4b) and IL17 level (*P* < 0.001, Fig. 4b) were obviously decreased in the miR130b-knockdown OCI-ly10 co-culture system, while remained constant in the miR130b-knockdown OCI-ly10 co-culture system upon treatment with OX40 agonistic antibody (Fig. 4b).

LNPs-miR130b control and LNPs-miR130b antagomir were also synthesized. The dynamic light scattering (DLS) result demonstrated particle size distributions of the LNPs, LNPs-miR130b control, and LNPs-miR130b antagomir (Supplementary Fig. 4a). Compared with the LNPs, the hydrodynamic diameter of LNPs-miR130b control and LNPs-miR130b antagomir slightly increased. The zeta potential of LNPs-miR130b control and LNPs-miR130b antagomir decreased significantly for the anionic miR130b control and miR130b antagomir weakened the positive charge of the LNPs (Supplementary Fig. 4a). The transmission electron microscope showed the spherical morphology of the LNPs, LNPs-miR130b control, and LNPs-miR130b antagomir (negatively stained by phosphotungstic acid) were less than 100 nm in diameter (Supplementary Fig. 4b). Cy5.5/Fam-labeled LNPs-miR130b control and Cy5.5/Fam-labeled LNPs-miR130b antagomir were utilized for in vitro experiments to investigate the uptake of the LNPs, miR130b control, and miR130b antagomir, which were mainly distributed in the cytoplasm and manifested with high endocytosis (Supplementary Fig. 4c). Cy5.5/Fam-labeled LNPs-miR130b antagomir showed significant knockdown efficiency in DB cells and OCI-ly10 cells, as compared to Cy5.5/Fam-labeled LNPs-miR130b control (*P* both <0.001, Supplementary Fig. 4d). Th17 cell percentage (*P* = 0.004, Fig. 4c) and IL17 level (*P* = 0.005, Fig. 4c) were significantly increased in the miR130b-overexpressing DB co-culture system upon treatment with LNPs-miR130b control, while reduced in the miR130b-overexpressing DB co-culture system upon treatment with LNPs-miR130b antagomir (*P* = 0.004 and *P* = 0.001, Fig. 4c). Also, Th17 cell percentage (*P* < 0.001, Fig. 4d) and IL17 level (*P* < 0.001, Fig. 4d) were remarkably decreased in the miR130b-knockdown OCI-ly10 co-culture system upon treatment with LNPs-miR130b control, while remained constant decreased in the miR130b-knockdown OCI-ly10 co-culture system upon treatment with LNPs-miR130b antagomir (Fig. 4d).

Moreover, we performed multi-color flow cytometry to examine the role of miR130b in immune cell subsets, including Th1, Th2, Th22, Treg, CD8 T cell, dendritic cell, macrophage, MDSC, and ELISA to detect related immunological cytokines including IFNγ, IL4, IL22, IL10, CXCL9, IL1β, MCP1, TNFα, CXCL8, IFNα, and IFNβ in the miR130b-overexpressing DB co-culture system and miR130b-knockdown OCI-ly10 co-culture system upon treatment with LNPs-miR130b control or LNPs-miR130b antagomir. No obvious change in the above immune cell subsets (Supplementary Fig. 5a, b) or related immunological cytokines (Supplementary Fig. 5c, d) was observed among the groups. Therefore, miR130b could increase Th17 cell percentage and promote IL17 production, which could be abrogated by LNPs-miR130b antagomir.

### OX40 agonistic antibody modulated B-lymphoma cell autophagy through OX40/OX40L-mediated lymphoma cell interaction with Th17 cells both in vitro and in vivo

**Fig. 5:**
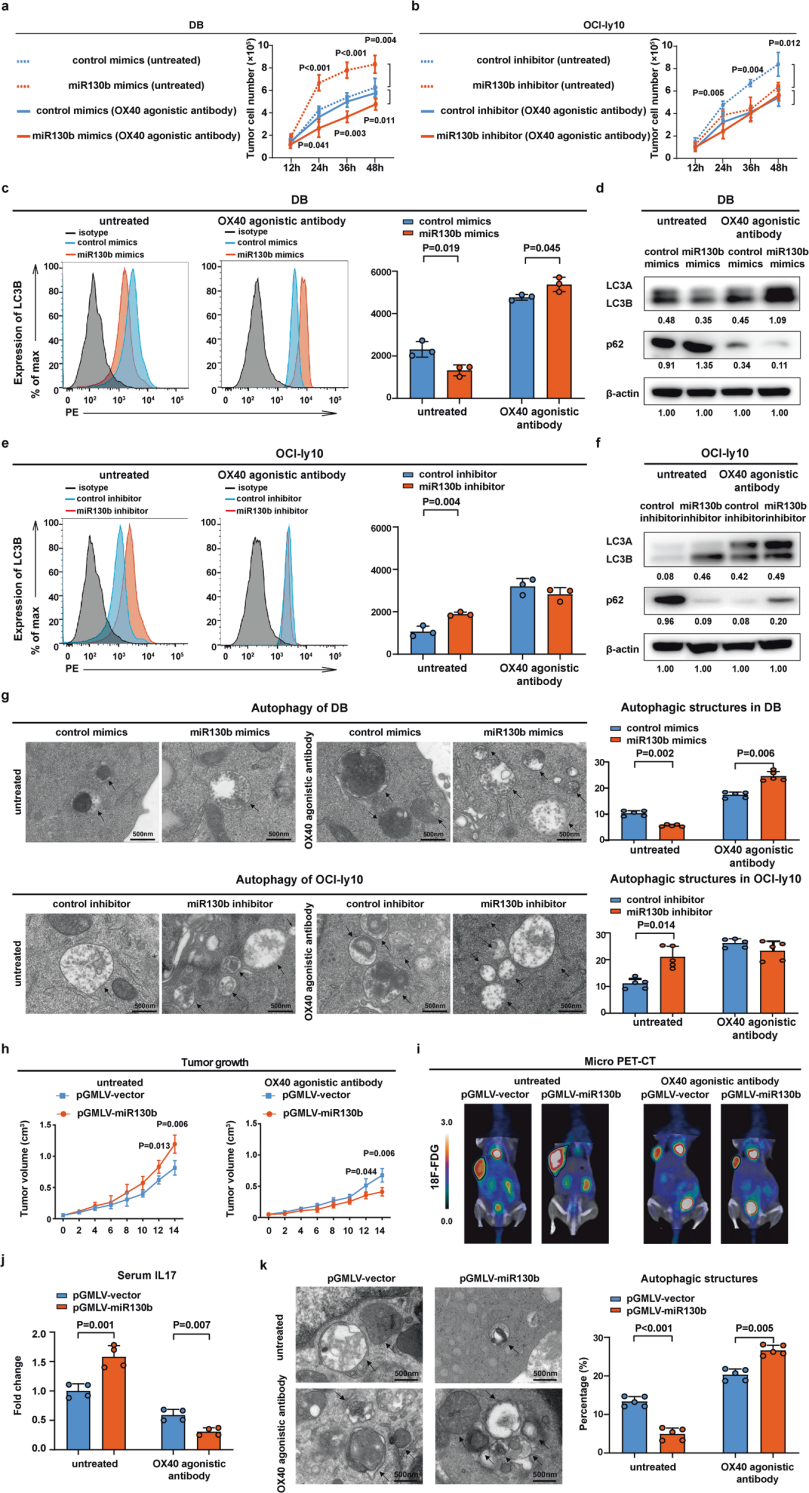
OX40 agonistic antibody modulated B-lymphoma cell autophagy through OX40/OX40L-mediated lymphoma cell interaction with Th17 cells both in vitro and in vivo. **a** Cell growth in the control mimics-transfected DB co-culture system and miR130b mimics-transfected DB co-culture system upon treatment with or without OX40 agonistic antibody. MTT assay was adopted to measure cell viability. Data are summarized as mean ± SD (*n* = 3). **b** Cell growth in the control inhibitor-transfected OCI-ly10 co-culture and miR130b inhibitor-transfected OCI-ly10 co-culture upon treatment with or without OX40 agonistic antibody. MTT assay was adopted to measure cell viability. Data are summarized as mean ± SD (*n* = 3). **c** Flow cytometry analysis of LC3B in the control mimics-transfected DB co-culture system and miR130b mimics-transfected DB co-culture system upon treatment with or without OX40 agonistic antibody. Data are summarized as mean ± SD (*n* = 3). **d** Western blot of LC3B and p62 in the control mimics-transfected DB co-culture system and miR130b mimics-transfected DB co-culture system upon treatment with or without OX40 agonistic antibody. **e** Flow cytometry analysis of LC3B in the control inhibitor-transfected OCI-ly10 co-culture and miR130b inhibitor-transfected OCI-ly10 co-culture upon treatment with or without OX40 agonistic antibody. Data are summarized as mean ± SD (*n* = 3). **f** Western blot of LC3B and p62 in the control inhibitor-transfected OCI-ly10 co-culture and miR130b inhibitor-transfected OCI-ly10 co-culture upon treatment with or without OX40 agonistic antibody. **g** Transmission electron microscope showed typical autophagosomes in the miR130b mimics-transfected DB co-culture system and miR130b inhibitor-transfected OCI-ly10 co-culture upon treatment with or without OX40 agonistic antibody. The cells were counted from five visions selected at random and subjected for statistical analysis. Data are summarized as mean ± SD (*n* = 5). **h** pGMLV-vector and pGMLV-miR130b cells were injected into nude mice subcutaneously with or without OX40 agonistic antibody treatment, and tumor volume was determined. Data are summarized as mean ± SD (*n* = 4). **i** Standardized uptake value intensity in pGMLV-vector group and pGMLV-miR130b group upon treatment with or without OX40 agonistic antibody were detected by micro PET-CT. **j** IL17 level was measured by ELISA in pGMLV-vector group and pGMLV-miR130b group upon treatment with or without OX40 agonistic antibody. Data are summarized as mean ± SD (*n* = 4). **k** Transmission electron microscope showed typical autophagosomes in pGMLV-vector group and pGMLV-miR130b group upon treatment with or without OX40 agonistic antibody. The cells were counted from five visions selected at random and subjected for statistical analysis. Data are summarized as mean ± SD (*n* = 5)

Cell growth was enhanced in the miR130b-overexpressing DB co-culture system (*P* < 0.001, *P* < 0.001, and *P* = 0.004, Fig. 5a), which was abrogated by OX40 agonistic antibody (*P* = 0.041, *P* = 0.003, and *P* = 0.011, Fig. 5a). Accordingly, cell growth was reduced in the miR130b-knockdown OCI-ly10 co-culture system (*P* = 0.005, *P* = 0.004, and *P* = 0.012, Fig. 5b), which was not altered by OX40 agonistic antibody (Fig. 5b). Without obvious change of cell apoptosis (Supplementary Fig. 6a, b) and cell cycle arrest (Supplementary Fig. 6c, d) among the groups, cell autophagy was further investigated. LC3B expression on DB cells was decreased in the miR130b-overexpressing DB co-culture system (*P* = 0.019, Fig. 5c), but upregulated upon treatment with OX40 agonistic antibody (*P* = 0.045, Fig. 5c). Meanwhile, the autophagic flux was confirmed by a lower expression of autophagosome-associated LC3B and higher p62 expression in the miR130b-overexpressing DB co-culture system. Meanwhile, OX40 agonistic antibody increased LC3B and decreased p62 expression in the miR130b-overexpressing DB co-culture system (Fig. 5d). A significant increase in LC3B was detected in the miR130b-knockdown OCI-ly10 co-culture system (*P* = 0.004, Fig. 5e), which was abrogated by OX40 agonistic antibody (Fig. 5e). The autophagic flux was also confirmed by higher LC3B expression and lower p62 expression in the miR130b-knockdown OCI-ly10 co-culture system, which were abrogated by OX40 agonistic antibody (Fig. 5f). Moreover, the ultrastructure of lymphoma cells was investigated in DB and OCI-ly10 cells sorted from the co-culture systems. Typical autophagosomes of lymphoma cells were observed frequently in the miR130b-overexpressing DB co-culture system of upon treatment with OX40 agonistic antibody (*P* = 0.002, *P* = 0.006, Fig. 5g, upper panel). However, no apparent change in typical autophagosomes was observed in the miR130b-knockdown OCI-ly10 co-culture system upon treatment with OX40 agonistic antibody (Fig. 5g, lower panel).

Serum IL17 acts as the main cytokine secreted by Th17 cells. The biological activities of Th17 cells equal to the expression levels of IL17.^20^ To determine the relationship between Th17 cells and tumor autophagy, cell growth was enhanced in the miR130b-overexpressing DB co-culture system (*P* = 0.033, *P* < 0.001 and *P* = 0.010, Supplementary Fig. 7a), which was abrogated in the miR130b-overexpressing DB co-culture system upon treatment with IL17 inhibitor (*P* = 0.016, Supplementary Fig. 7a). Accordingly, cell growth was reduced in the miR130b-knockdown OCI-ly10 co-culture system (*P* < 0.001, Supplementary Fig. 7b), which was not altered in the miR130b-knockdown OCI-ly10 co-culture system upon treatment with IL17 inhibitor (Supplementary Fig. 7b). Typical autophagosomes of lymphoma cells were observed frequently in the miR130b-overexpressing DB co-culture system upon treatment with IL17 inhibitor (*P* = 0.002, *P* = 0.001, Supplementary Fig. 7c upper panel). However, no apparent change in typical autophagosomes was observed in the miR130b-knockdown OCI-ly10 co-culture system upon treatment with IL17 inhibitor (*P* < 0.001, Supplementary Fig. 7c, lower panel).

A20 cells stably transfected with pGMLV-vector or pGMLV-miR130b were subcutaneously injected to establish murine xenograft model. Compared with pGMLV-vector tumors (*P* = 0.013 at day 12, *P* = 0.006 at day 14, Fig. 5h), OX40 agonistic antibody exhibited an effective anti-tumor activity on pGMLV-miR130b tumors (*P* = 0.044 at day 12, *P* = 0.006 at day 14, Fig. 5h). Tumors implanted in the flank of mice were visualized by f-fluorodeoxyglucose of small-animal PET/CT (Fig. 5i). Compared with the pGMLV-vector group, serum IL17 was significantly increased in the pGMLV-miR130b group, which was inhibited upon treatment with OX40 agonistic antibody (*P* = 0.001, *P* = 0.007, Fig. 5j). Accordingly, compared with the pGMLV-vector group, lymphoma cell autophagy was decreased in the pGMLV-miR130b group, whereas enhanced upon treatment with OX40 agonistic antibody (*P* < 0.001, *P* = 0.005, Fig. 5k). Therefore, miR130b could inhibit B-lymphoma cell autophagy, which was counteracted by OX40 agonistic antibody.

### LNPs-miR130b antagomir exhibited in vitro and in vivo activity on OX40-impaired B-cell lymphoma

**Fig. 6:**
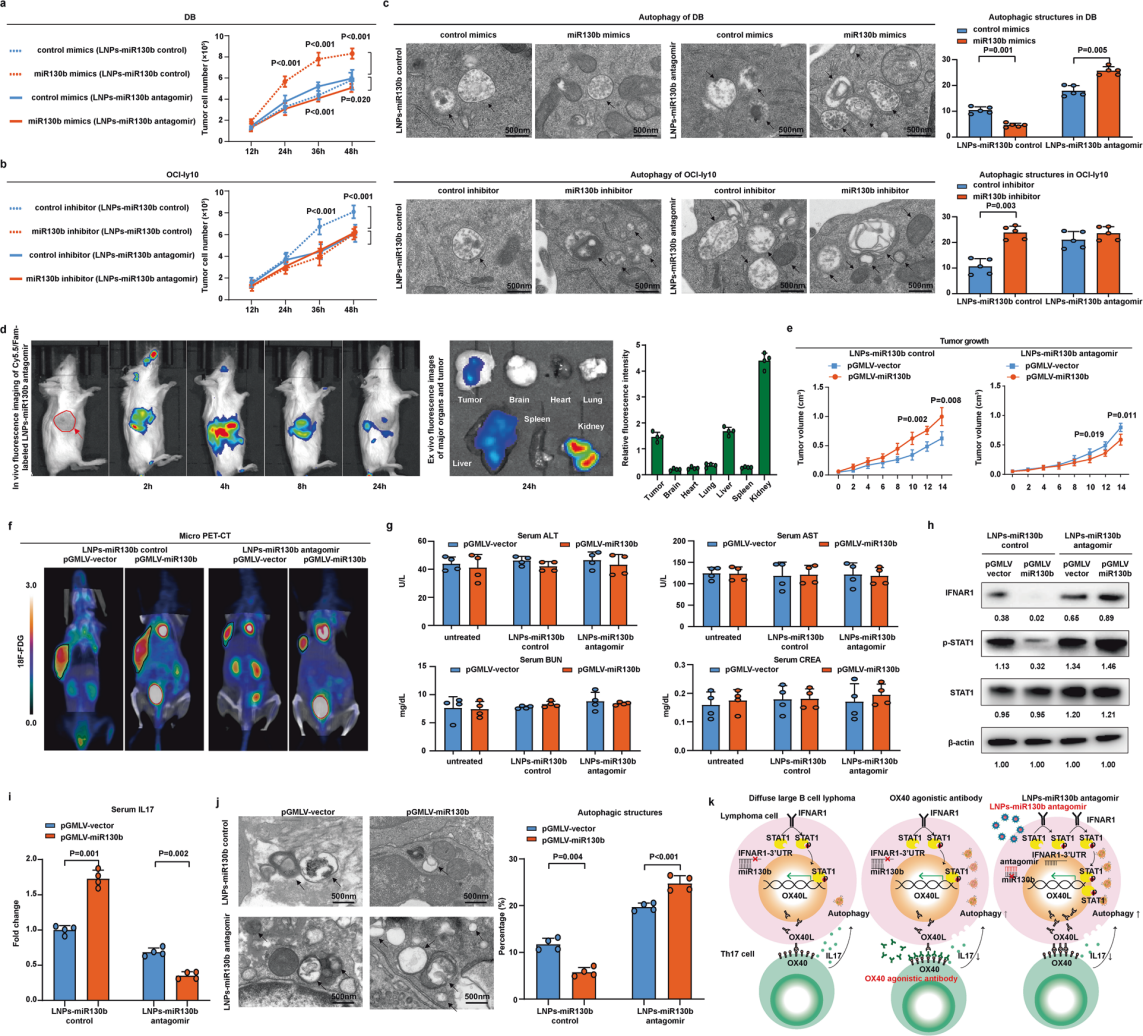
LNPs-miR130b antagomir exhibited in vitro and in vivo activity on OX40-impaired B-cell lymphoma. **a** Cell growth in the control mimics-transfected DB co-culture system and miR130b mimics-transfected DB co-culture system upon treatment with LNPs-miR130b control or with LNPs-miR130b antagomir. MTT assay was adopted to measure cell viability. Data are summarized as mean ± SD (*n* = 3). **b** Cell growth in the control inhibitor-transfected OCI-ly10 co-culture and miR130b inhibitor-transfected OCI-ly10 co-culture upon treatment with LNPs-miR130b control or with LNPs-miR130b antagomir. Cell viability was measured by MTT. Data are summarized as mean ± SD (*n* = 3). **c** Transmission electron microscope showed typical autophagosomes in the miR130b mimics-transfected DB co-culture system and miR130b inhibitor-transfected OCI-ly10 co-culture upon treatment with LNPs-miR130b control or LNPs-miR130b antagomir. The cells were counted from five visions selected at random and subjected for statistical analysis. Data are summarized as mean ± SD (*n* = 5). **d** Standardized uptake value intensity of Cy5.5/Fam-labeled LNPs-miR130b antagomir after intravenous injection (left panel) were measured by micro PET-CT. The column indicated ex vivo fluorescence images of the tumor and major organs, including brain, heart, lung, liver, spleen, and kidney, from mice at 24 h after intravenous injection (right panel). **e** pGMLV-vector and pGMLV-miR130b cells were injected into nude mice subcutaneously with LNPs-miR130b control or LNPs-miR130b antagomir treatment, and tumor volume was determined. Data are summarized as mean ± SD (*n* = 4). **f** Micro PET-CT showed standardized uptake value intensity in pGMLV-vector group and pGMLV-miR130b group upon treatment with LNPs-miR130b control or LNPs-miR130b antagomir. **g** Serum ALT, AST, and Serum BUN, CREA were measured using a clinical chemistry analyzer in pGMLV-vector group and pGMLV-miR130b group between untreated group and treatment with LNPs-miR130b control group or LNPs-miR130b antagomir group. Data are summarized as mean ± SD (*n* = 4). **h** Western blot analysis of IFNAR1, p-STAT, STAT1 in pGMLV-vector group and pGMLV-miR130b group upon treatment with LNPs-miR130b control or LNPs-miR130b antagomir. **i** IL17 level was measured by ELISA in pGMLV-vector group and pGMLV-miR130b group upon treatment with LNPs-miR130b control or LNPs-miR130b antagomir. Data are summarized as mean ± SD (*n* = 4). **j** Transmission electron microscope showed typical autophagosomes in pGMLV-vector group and pGMLV-miR130b group upon treatment with LNPs-miR130b control and LNPs-miR130b antagomir. The cells were counted from five visions at random and subjected for statistical analysis. Data are summarized as mean ± SD (*n* = 5). **k** Graphic working model illustrated that miR130b counteracts diffuse large B-cell lymphoma progression via OX40/OX40L-mediated interaction with Th17 cells

To determine whether LNPs-miR130b antagomir could also be an alternative immunotherapeutic strategy for OX40 agonistic antibody, we treated the co-culture systems with LNPs-miR130b control and LNPs-miR130b antagomir. DB cell growth was increased in the miR130b-overexpressing DB co-culture system upon treatment with LNPs-miR130b control (*P* all <0.001, Fig. 6a), whereas downregulated upon treatment with LNPs-miR130b antagomir (*P* < 0.001, *P* = 0.020, Fig. 6a). OCI-ly10 cell growth was inhibited in the miR130b-knockdown OCI-ly10 co-culture system upon treatment with LNPs-miR130b control (*P* both <0.001, Fig. 6b). However, no obvious difference in cell growth inhibition was detected when treated with LNPs-miR130b antagomir (Fig. 6b). Moreover, the ultrastructure of lymphoma cells was investigated in DB and OCI-ly10 cells sorted from the co-culture systems. Typical autophagosomes of lymphoma cells were observed frequently in the miR130b-overexpressing DB co-culture system upon treatment with LNPs-miR130b antagomir (*P* = 0.001, *P* = 0.005, Fig. 6c, upper panel). However, no apparent change in typical autophagosomes was observed in the miR130b-knockdown OCI-ly10 co-culture system upon treatment with LNPs-miR130b antagomir (Fig. 6c, lower panel).

The fluorescence intensity manifested that Cy5.5/Fam-labeled LNPs-miR130b antagomir was significantly enriched in the tumors, suggesting potential therapeutic efficacy (Fig. 6d). The Cy5.5/Fam-labeled LNPs-miR130b antagomir was also concentrated in the livers and kidneys responsible for drug excretion (Fig. 6d). The murine xenograft model was established as described before. Compared with LNPs-miR130b control (*P* = 0.002 at day 12, *P* = 0.008 at day 14, Fig. 6e), LNPs-miR130b antagomir exhibited an effective anti-tumor activity on pGMLV-miR130b tumors (*P* = 0.019 at day 12, *P* = 0.011 at day 14, Fig. 6e). F-fluorodeoxyglucose small-animal PET/CT was used to visualize implanted tumors in the flank of mice (Fig. 6f). Liver function (serum alanine aminotransferase, ALT and aspartate aminotransferase, AST) and kidney function (serum urea nitrogen, BUN and creatinine, CREA) were measured. No significant difference was observed in liver function and kidney function between pGMLV-vector group and pGMLV-miR130b group, among untreated group, LNPs-miR130b control group, and LNPs-miR130b antagomir group (Fig. 6g). Compared with the pGMLV-vector group, IFNAR1 and p-STAT1 expression were decreased in the pGMLV-miR130b group upon treatment with LNPs-miR130b control, while increased upon treatment with LNPs-miR130b antagomir (Fig. 6h). IL17 secretion was significantly increased in the pGMLV-miR130b group upon treatment with LNPs-miR130b control (*P* = 0.001, Fig. 6i), while inhibited upon treatment with LNPs-miR130b antagomir (*P* = 0.002, Fig. 6i). Accordingly, compared with the pGMLV-vector group, lymphoma cell autophagy was decreased in the pGMLV-miR130b group upon treatment with LNPs-miR130b control (*P* = 0.004, Fig. 6j), whereas enhanced upon treatment with LNPs-miR130b antagomir (*P* < 0.001, Fig. 6j). In summary, miR130b enhanced B-lymphoma cell sensitivity to OX40 agonistic antibody and LNPs miR130b antagomir through modulating OX40/OX40L-mediated lymphoma cell interaction with Th17 cells via IFNAR1/p-STAT1 axis (Fig. 6k).

## Discussion

MiRNAs are critically involved in lymphoma development and progression by regulating the tumor microenvironment. MiR130b could act as communicators between tumor cells and microenvironmental cells.^22,23^ Indeed, we demonstrated a significant correlation of serum miR130b with tumor miR130b in DLBCL, and verified the role of miR130b on poor prognosis and lymphoma relapse in a large cohort of patients. Serum IL17, the main cytokine secreted by Th17 cells, was accordingly increased, further indicating that miR130b was biologically functional. Th17 cells and IL17 possess tumor promoting effects through stimulating tumor cell stemness, proliferation, migration, and invasion. In addition, Th17 cells can modulate immune cells, endothelial cells and stromal cells, inducing an immunosuppressive tumor microenvironment.^24^ In DLBCL, Th17 cells were reported to provoke rituximab resistance by suppressing p53 expression and inhibiting cell apoptosis.^25^ To our knowledge, this study provides direct evidence that serum miR130b contributed to DLBCL progression through Th17-asscociated immunosuppressive microenvironment.

IFNAR1 downregulation contributes to the suppressive tumor microenvironment. Stabilizing IFNAR1 inhibits tumor growth and improves immunotherapy efficacy.^26^ The cytoplasmic domain of IFNAR1 is required for the phosphorylation of STAT1 at tyrosine-701 and subsequent STAT1 expression.^21^ IFNAR1 induces p-STAT1 and tumor immunity through modulating downstream immune checkpoint genes.^27^ Moreover, OX40L expression is dependent on p-STAT1 to reduce Th17 cell number and function in autoimmune diseases.^28,29^ Here, we showed that IFNAR1/p-STAT1 axis was involved in OX40/OX40L-mediated B-lymphoma cell interaction with Th17 cells in DLBCL. OX40 enhances immunological responses against tumor cells through binding OX40L, such as lung cancer, melanoma and Hodgkin’s lymphoma.^6,30^ OX40/OX40L interaction inhibits Th17 cell growth and IL17 secretion in vitro and in vivo.^31^ OX40 agonistic antibody can abrogate Th17 cell proliferation, decrease IL17 level, and enhance tumor immunity.^32^ Clinically, OX40 agonistic antibody is effective in treating patients with advanced solid tumors, accompanied by increased peripheral T-cell activation.^33^ However, the OX40 agonistic antibody is commonly associated with systemic toxicity.^7^ Consistent with the previous study that miR130b overexpression inhibits signaling downstream from the type I IFN pathway,^34^ we showed that miR130b downregulated tumor OX40L expression via type I IFN receptor IFNAR1 in DLBCL, thereby provoked Th17 accumulation, and induced immunosuppressive status in an OX40/OX40L-dependent manner. More interestingly, as an oncogenic biomarker in the tumor microenvironment, miR130b leads to sensitization of B-lymphoma cells to OX40 agonistic antibody. OX40/OX40L blockade downregulated multiple genes involved in autophagy.^35^ Accumulating evidences support that autophagy promotes cell death through degradation of anti-apoptotic and cell survival factors in cancer.^36^ In DLBCL, patients with high Beclin1 expression achieve better OS, PFS, and overall response rate to R-CHOP.^37^ Furthermore, autophagy also represents a promising strategy of cancer treatment. EZH2 inhibitors such as GSK126 and EPZ-011989 induce autophagy^38^ and manifest significant tumor cell growth inhibition in DLBCL.^39^

With increasing interests in miRNA therapeutics, miRNA-based drugs are being innovatively tested in clinical trials for cancer patients.^40^ A Phase I trial (NCT02580552ix) was conducted to evaluate the safety and efficacy of miR155 antagomir in patients with hematological cancers, particularly DLBCL, and showed a sustained reduction in tumor burden with an acceptable safety profile.^41,42^ Another Phase II trial (NCT01200420) revealed long-term safety and effect of oncogenic miR122 antagomir in liver cancer patients.^43^ LNPs are currently demonstrated as potential RNA-delivering vehicles targeting tumor cells, providing maximized therapeutic efficiency and minimized systemic effects.^44^ Here, we constructed novel lipid nanoparticles to deliver miR130b antagomir into B-lymphoma cells and confirmed the inhibitory effect of LNPs-miR130b antagomir on B-lymphoma cells both in vitro and in vivo.

In conclusion, as an oncogenic biomarker of DLBCL, miR130b was related to lymphoma progression through modulating OX40/OX40L-mediated lymphoma cell interaction with Th17 cells, attributing to B-cell lymphoma sensitivity towards OX40 agonistic antibody. Targeting miR130b using LNPs-miR130b antagomir could also be a potential immunotherapeutic strategy in treating OX40-altered lymphoid malignancies.

## Materials and methods

### Patients

This study enrolled 532 patients with newly diagnosed DLBCL receiving standard immunochemotherapy rituximab, cyclophosphamide, doxorubicin, vincristine, and prednisone (R-CHOP) between August 2012 and April 2018. According to World Health Organization (WHO) classification, histological diagnosis was established. The Institutional Review Boards approved the study with informed consent obtained from all patients in accordance with the Declaration of Helsinki.

### Cells and reagents

DB (Human B-lymphoma cell line) and A20 (murine B-lymphoma cell line) from American Type Culture Collection (Manassas, VA, USA) were obtained, and OCI-ly10 was kindly provided by Huang CX. DB and A20 were grown in RPMI-1640 medium, and OCI-ly10 was grown in an IMDM medium with 10% heat-inactivated fetal bovine serum (FBS) added in a humidified atmosphere containing 5% CO_2_ at 37°. PBMCs were isolated from a healthy donor using ficoll density gradient centrifugation. To purify PBMCs, the interface of ficoll medium was carefully removed, washed twice with salt-buffered solution, then centrifuged at 300 × *g* for 5 min. Both p-STAT1 inhibitor (S1491) and IL17 inhibitor (A2025) were obtained from selleck (Houston, TX, USA).

### Serum and tissue miR130b assessment

MiRNeasy Serum/Plasma Kit (Qiagen, Valencia, CA, USA) was used to extract total serum miRNA. MiR130b expression was calculated by quantitative real-time PCR using MiScript Reverse Transcription Kit (Qiagen), miR130b primer (MS00008610, Qiagen), and MiScript SYBR Green PCR Kit (Qiagen). Endogenous control was miR39 (MS00019789, Qiagen) and calibration was DB cells. Trizol agent (Invitrogen, Carlsbad, CA, USA) was used to extract total tissue miRNA. Endogenous control was RNU6 (MS00033740, Qiagen) and calibration was DB cells. 7500HT Fast Real-time PCR system (Applied Biosystem, Carlsbad, CA, USA) was used to analyze the reactions. ^2−ΔΔ^CT method was used to calculate the relative quantification.

### Enzyme-linked immunosorbent assay (ELISA)

Human serum IL17 (HS170), IFNγ (DIF50C), IL4 (D4050), IL22 (D2200), IL10 (D1000B), CXCL9 (DCX900), IL1β (DLB50), MCP1 (DCP00), TNFα (DTA00D), CXCL8 (D8000C), IFNα (41100-1), and IFNβ (DIFNB0) were detected using Human ELISA Kit (R&D Systems, Minneapolis, MN, USA) according to the manufacturer’s operating instructions. Murine serum IL17 was measured using Mouse IL17 ELISA Kit (M1700, R&D Systems) following the manufacturer’s protocol.

### In vitro co-culture system

Transwell cell culture chambers (8 μM, Millipore Corporation, Billerica, MA, USA) were used for the co-culture assay. PBMCs are derived from peripheral blood of healthy volunteer, with a mixed population of myeloid and lymphoid cells including B cells (~15%), T cells (~70%), monocytes (~5%), and natural killer (NK) cells (~10%).^45^ Effector (E) to target (T) ratio is defined as ratio of number of PBMCs to lymphoma cells. The *E*:*T* ratio was 5:1, as previously suggested.^46^ In the 8 μM co-culture system, the upper chamber was placed with lymphoma cells and the lower chamber was placed with PBMCs. With the cells in lower chamber collected, lymphoma cells were sorted by EasySepTM Human CD20+ Cell Isolation Kit, and Th17 cells were sorted by EasySepTM Human Th17 Cell Enrichment Kit.

### Cell transfection

DB cells were transfected with control mimics (Riobio, Guangzhou, China) or miR130b mimics (Riobio) at 100 nm using lipofectamine 2000 (Invitrogen) for 24 h following the manufacturer’s protocol. For the knockdown assay, OCI-ly10 cells were transfected with control inhibitor (Riobio) or 100 nm miR130b inhibitor (Riobio) at 100 nm using lipofectamine 2000 for 24 h.

### Lentivirus packaging and transfection

To knockdown IFNAR1, STAT1, or OX40L in DB cells, purified plasmid pLKO.1-shIFNAR1 (IFNAR1 KO), pLKO.1-shSTAT1 (STAT1 KO), pLKO.1-shOX40L (OX40L KO), or pLKO.1-vector (KO control) was constructed. To overexpress IFNAR1, STAT1, or OX40L in OCI-ly10 cells, purified plasmid MIGR1-IFNAR1 (IFNAR1 over), MIGR1-STAT1 (STAT1 over), MIGR1-OX40L (OX40L over) or MIGR1-vector (over control) was constructed. To delete the cytoplasmic domain 486aa to 511aa of IFNAR1, purified plasmid IFNAR1^△486–511^ or full-length IFNAR1 (IFNAR1^FL^) was constructed. To obtain point mutation tyrosine-701 of STAT1, purified plasmid MIGR1-STAT1^Y701F^ and MIGR1-STAT1^WT^ plasmid was constructed. Above plasmids were transfected into HEK-293T cells with package vectors using lipofectamine 2000 (Invitrogen). The HEK-293T cell culture supernatant was condensed into a viral concentration of approximately 3 × 10^8^ transducing units/ml. The lentiviral vector particles were incubated overnight with DB cells or OCI-ly10 cells. The stably transfected cells were isolated upon treatment with 4 μg/ml puromycin and stable clones were selected upon treatment with 1 μg/ml puromycin or obtained by green fluorescence protein (GFP). To overexpress miR130b in A20 cells, either purified plasmids pGMLV-vector or pGMLV-miR130b were transfected into HEK-293T using lipofectamine 2000 (Invitrogen). The HEK-293T cell culture supernatant was condensed into a viral concentration of approximately 3 × 10^8^ transducing units/ml. The lentiviral particles were incubated overnight with A20 cells. Stably transfected cells were obtained by GFP.

### Luciferase report assay

To amplify the 3′UTR (1646-1652 bp) of IFNAR1, total cDNA from HEK-293T cells was utilized, forward primer: 5′-GATCGCCGTGTAATTCTAGAATTACCCATGGATATCCTTAATAG-3′; reverse primer: 5′-CCGGCCGCCCCGACTCTAGACCTTTTCTTCCACCAGGATCATC-3′. The XbaI restriction enzyme site was used. HEK-293T cells were plated in 24-well plates and co-transfected with either control mimics (100 nM) or miR130b mimics (100 nM), either IFNAR1 wild-type (IFNAR1^WT^, 100 ng/ml) or IFNAR1 mutant (IFNAR1^MUT^, 100 ng/ml) luciferase reporter construct, and luciferase reporter (10 ng/ml) using lipofectamine 2000. To amplify the promoter (−1130 to −1140bp) of OX40L, total cDNA from HEK-293T cells was utilized, forward primer: 5′-CGATAGGTACCGAGCTCTTACGCGTATGAAGAACTATTACACAAGAATTGGGC-3′; reverse primer: 5′-GCTTACTTAGATCGCAGATCTCGAGAAAGCGGACTCTCTCAGTTTTAACG-3′. The MluI and XboI restriction enzyme site were used. HEK-293T cells were plated in 24-well plates and co-transfected with either MIGR1-vector (1 µg/ml) or MIGR1-STAT1 (1 µg/ml), either OX40L wild-type (OX40L^WT^, 0.25 µg/ml) or OX40L mutant (OX40L^MUT^, 0.25 µg/ml) luciferase reporter construct, and luciferase reporter (10 ng/ml) using lipofectamine 2000. Cells were lysed using the Passive Lysis Buffer (30 μl per well) from Dual-Luciferase Reporter Assay System Kit (Promega, Madison, WI, USA). Lysates were examined directly by Centro XS3 LB960 Luminometer (Berthold, Bad Wildbad, Germany) using the Dual-Luciferase Reporter Assay System.

### Quantitative real-time PCR

Total RNA was extracted from B-lymphoma cells using Trizol reagent and reverse transcribed into first-strand cDNA using a PrimeScript RT Reagent Kit (RR047A, TaKaRa, Shiga, Japan). Quantitative real-time PCR was performed on ABI ViiA7 (Applied Biosystems) using SYBR Premix Ex Taq TM II kit (RR820A, TaKaRa). Forward primer of IFNAR1: 5′-AACAGGAGCGATGAGTCTGTC-3′; Reverse primer: 5′-TGCGAAATGGTGTAAATGAGTCA-3′. Forward primer of IFNAR2: 5’-TCATGGTGTATATCAGCCTCGT-3′; Reverse primer: 5′-AGTTGGTACAATGGAGTGGTTTT-3′. Forward primer of STAT1: 5′-CAGCTTGACTCAAAATTCCTGGA-3′; Reverse primer: 5′-TGAAGATTACGCTTGCTTTTCCT-3′. Forward primer of STAT2: 5′-CCAGCTTTACTCGCACAGC-3′; Reverse primer: 5′-AGCCTTGGAATCATCACTCCC-3′. Forward primer of STAT3: 5′-CAGCAGCTTGACACACGGTA-3′; Reverse primer: 5′-AAACACCAAAGTGGCATGTGA-3′. Forward primer of OX40L: 5′-CCAGGCCAAGATTCGAGAGG-3′; Reverse primer: 5′-CCGATGTGATACCTGAAGAGCA-3′. Forward primer of GAPDH: 5′-GGAGCGAGATCCCTCCAAAAT-3′; Reverse primer: 5′-GGCTGTTGTCATACTTCTCATGG-3′. ^2−∆∆^CT methods was used to calculate the relative quantification.

### Multi-color flow cytometry

OX40L expression on B-lymphoma cells was assessed using anti-CD19 (555415, Becton Dickinson, Franklin Lakes, NJ, USA), anti-OX40L (563473, Becton Dickinson). Immune checkpoint gene expression on B-lymphoma cells including PD-L1, CD80, 4-1BBL, LAG3, TIGHT, TIM3, and VISTA was assessed using anti-PD-L1 (751185, Becton Dickinson), anti-CD80 (624352, Becton Dickinson), anti-4-1BBL (624294, Becton Dickinson), anti-LAG3 (624350, Becton Dickinson), anti-TIGHT (747846, Becton Dickinson), anti-TIM3 (35-3109-42, Invitrogen), anti-VISTA (624296, Becton Dickinson) and APC Mouse IgG1 κ was used as isotype control (554681, Becton Dickinson). For Th17 cell detection, co-cultured cells were stained with anti-CD4 (560649, Becton Dickinson) and anti-RORγt (563282, Becton Dickinson). For Th1 cell detection, co-cultured cells were stained with anti-CD4 (624298, Becton Dickinson) and anti-T-Bet (624295, Becton Dickinson). For Th2 cell detection, co-cultured cells were stained with anti-CD4 (624298, Becton Dickinson) and anti-GATA3 (560405, Becton Dickinson).^47^ For Th22 cell detection, co-cultured cells were stained with anti-CD4 (624298, Becton Dickinson), anti-CCR4 (624348, Becton Dickinson) and anti-CCR6 (566130, Becton Dickinson). For Treg cell detection, co-cultured cells were stained with anti-CD4 (624298, Becton Dickinson) and anti-Foxp3 (624279, Becton Dickinson). For CD8 T cell detection, co-cultured cells were stained with anti-CD8 (563919, Becton Dickinson). For dendritic cell and MDSC detection, co-cultured cells were stained with anti-CD11b (562632, Becton Dickinson) and anti-HLA-DR (612980, Becton Dickinson). For macrophage detection, co-cultured cells were stained with anti-CD11b (562632, Becton Dickinson) and anti-CD68 (624380, Becton Dickinson). Cell autophagy was assessed using anti-LC3B (13611S, Cell Signaling Technology, Danvers, MA, USA), PE Rabbit IgG was used as isotype control (ab37407, Abcam, Cambridge, United Kingdom). Cell cycle analysis was detected by Cell Cycle Kit (558662, Becton Dickinson). Cell apoptosis was analyzed using ANXA5/Annexin V-Phycoerythrin Kit (559763, Becton Dickinson). Data was acquired using BD FACS Canto II flow cytometer (Becton Dickinson) and further analyzed using FlowJo software.

### Immunofluorescence assay

Methanol-fixed cells for immunofluorescence assay were stained using antibodies against OX40L (1:100, ab264466, Abcam, Cambridge, UK) and OX40 (1:100, AF3388, R&D Systems). Goat anti-mouse IgG, FITC-conjugate (1:100, ab6785, Abcam) (1:100, ab150075, Abcam) and donkey anti-rabbit IgG, Texas red conjugate were used as the secondary antibody. The fluorescence intensity of OX40L, OX40, and OX40/OX40L interaction were quantified using the Image J software by analyzing the ratio of the fluorescence values of OX40L or OX40 positive cells/the fluorescence values of total cells × 100%.

### Immunohistochemistry

Five micrometer-paraffin sections were applied to perform using antibodies against RORγt (1:1000, ab207082, Abcam) following the manufacturer’s protocol. RORγt expression levels were evaluated semi-quantitatively in terms of percentage of positive cells: − (no expression of RORγt), + (1–10% expression of RORγt), ++ (11–50% expression of RORγt) and +++ (more than 50% expression of RORγt).^48^

### Western blot

DB and OCI-ly10 were isolated and lysed in lysis buffer (200 μl) (Sigma Aldrich, Shanghai, China). Protein lysates (10 μg) were electrophoresed on 10% sodium dodecyl sulfate-polyacrylamide gel electrophoresis (SDS-PAGE) and transferred to nitrocellulose membranes (BioRad, Hercules, CA, USA). Membranes were blocked for non-specific binding using 5% non-fat dried milk and left overnight at 4 °C rocking at low speed with IFNAR1 (ab45172, Abcam), IFNAR2 (ab190664, Abcam), p-STAT1 (9167, Cell Signaling Technology), STAT1 (14994 Cell Signaling Technology), p-STAT2 (4108, Cell Signaling Technology), STAT2 (72604, Cell Signaling Technology), p-STAT3 (9145, Cell Signaling Technology), STAT3 (9139, Cell Signaling Technology), OX40L (ab264466, Abcam), LC3A/B (4108, Cell Signaling Technology), p62 (88588, Cell Signaling Technology), and β-actin primary antibody (3700, Cell Signaling Technology). Horseradish peroxidase conjucated antibody was used as secondary detection. Immunocomplexes were visualized with an enhanced chemiluminescence detection kit.

### Transmission electron microscopy

Cell and tissue samples were fixed in glutaraldehyde (2%) at 4 °C overnight, washed in cacodylate buffer (0.1 M), postfixed in osmium tetroxide (1%) for 2 h at 4 °C, dehydrated through a graded ethanol series and embedded in plastic resin. Ultrathin sections (60–100 nm) were collected on cop per grids, stained with uranyl acetate (2%) and lead citrate (0.1%), and viewed with a transmission electron microscope (Philips CM120, Amsterdam, Netherlands). Ultrastructural hallmarks focused on double-membrane autophagic vesicles named autophagosomes, which was a gold standard for autophagy and autolysosomes. Cell autophagy was quantified by estimating the cell volume fractions occupied by autophagic structures using ImageJ software (20 cell profiles per sample), according to the guidelines for monitoring autophagy.^49^

### Synthesis of the antagomir loaded LNPs

Dextran was dissolved in water, kept under N2 protection. Later, the initiator ceric ammonium nitrate was added and stirred for 5 min, then the monomer methyl acrylate was added into the solution. Thirty minutes after the addition of the monomer, the crosslinker diallyl disulfide was added into the system. Subsequently, the sample was purified via dialysis against distilled water for 3 days to yield the NGs. 1,2-dioleoyl-3-trimethyl-ammonium-propane (DOTAP) and dioleoyl phosphatidylethanolamine (DOPE) were dissolved in chloroform. The solvent was then removed using rotary evaporation, and 5 ml dextran nanogels were added and further sonicated to form the LNPs.

LNPs-miR130b control and LNPs-miR130b antagomir were prepared by mixing LNPs (80 mg/ml) and miR130b control (20 μM) or miR130b antagomir (20 μM) at equal volumes, and the resultant mixture was subsequently vortexed for 20 s at room temperature. Cy5.5/Fam-labeled LNPs-miR130b control and Cy5.5/Fam-labeled LNPs-miR130b antagomir were prepared by mixing Cy5.5-labeled LNPs (80 mg/ml) and Fam-labeled miR130b control (20 μM) or Fam-labeled miR130b antagomir (20 μM) at equal volumes, and the resultant mixture was subsequently vortexed for 20 s at room temperature.

### Characterization of LNPs-miR130b control and LNPs-miR130b antagomir

The dynamic light scattering, hydrodynamic size, and zeta potential of LNPs, LNPs-miR130b control, and LNPs-miR130b antagomir were performed at a concentration of 1 mg/ml using Zetasizer Nano ZS90 instrument (Malvern Panalytical Ltd, Malvern, United Kingdom).

### Tracking of Cy5.5/Fam-labeled LNPs-miR130b control and Cy5.5/Fam-labeled LNPs-miR130b antagomir

Uptake of Cy5.5/Fam-labeled LNPs-miR130b control and Cy5.5/Fam-labeled LNPs-miR130b antagomir was visualized by the confocal microscope Zeiss LSM 880 Airyscan Elyra PS.1 (Zeiss, Oberkochen, Germany).

### RNA-sequencing

Tumor samples of 124 patients were analyzed using RNA-sequencing for gene expression profiles. The RNA library was constructed using TruSeq RNA Sample Preparation Kit. The cDNA library clusters were generated and sequenced on HiSeq 2000 system using TruSeq SBS Kit. Paired-end sequencing was performed using Illumina Hiseq X10. T. Gene expression levels in RNA-sequencing were estimated as previously described.^14^

### Tumor immunophenotype analysis

To investigate tracking tumor immunophenotype (TIP, http://biocc.hrbmu.edu.cn/TIP/index.jsp) analysis, RNA-sequencing data was analyzed. Based on the cancer-immunity cycle and inferring the tumor-infiltrating immune cells proportion, profiling the immune microenvironment was highlighted. The input was Transcripts Per Kilobase of exon model per Million mapped reads (TPM) values, and 178 signature genes (including stimulatory and inhibitory genes, grouped into 23 sets) were used as previously published.^50^ The final score was identified by examining the difference between the normalized scores of the stimulatory gene set and the inhibitory gene set for each sample.

### Murine model

Six weeks-old BALB/c mice (Shanghai Laboratory Animal Center, Shanghai, China) were subcutaneously injected into the right flank with 1 × 10^6^ A20 cells. Treatments initiated when tumors were approximately 0.5 cm × 0.5 cm in surface (Day 0). Invivomab anti-mouse OX40 agonistic antibody (BE0031, Bioxcell, NH, USA) was injected 200 μg per mouse. LNPs-miR130b control and LNPs-miR130b antagomir was injected at a dose of 12.5 mg per kg of body weight, three times per week for 2 weeks. Tumor volumes of the mice were calculated by the formula: volume = 0.5 × *a* (length) × *b* (width).^2^ Murine models were injected with 100 μl Cy5.5/Fam-labeled LNPs-miR130b antagomir to study the in vivo biodistribution. Fluorescence imaging of Cy5.5/Fam-labeled LNPs-miR130b antagomir was conducted at indicated time points after intravenous injection utilizing in vivo IVIS Lumina II optical imaging system. Animals were used according to the protocols approved by the Shanghai Rui Jin Hospital Animal Care and Use Committee.

### Statistical analysis

Mann–Whitney *U*-test was performed to calculate miR130b expression variance among groups. PFS was assessed from the start date of treatment and ended with the date of disease progression or the last follow-up. OS was recognized from start date of diagnosis and ended with the date of death or the last follow-up. Experimental results in vitro were calculated to compare variance by *t*-test and summarized as mean ± SD of three separate experiments. All statistical procedures were conducted with GraphPad Prism 8 software or SPSS version 20.0 statistical software package. *P* value less than 0.05 was regarded as statistically significant.

## Supplementary information

Therapeutic targeting miR130b counteracts diffuse large B-cell lymphoma progression via OX40/OX40L-mediated interaction with Th17 cells

## Data Availability

The data sets used and/or analyzed during the current study are available from the corresponding author on reasonable request.
