# Supplementary material for: Therapeutic targeting miR130b counteracts diffuse large B-cell lymphoma progression via OX40/OX40L-mediated interaction with Th17 cells
Source: Signal Transduct Target Ther. 2022 Mar 18;7:80. doi: 10.1038/s41392-022-00895-2 (PMC8931122; doi:10.1038/s41392-022-00895-2)
Supplement: Supplementary file 1 — Therapeutic targeting miR130b counteracts diffuse large B-cell lymphoma progression via OX40/OX40L-mediated interaction with Th17 cells [file 41392_2022_895_MOESM1_ESM.pdf]

Supplementary Materials for

Therapeutic targeting miR130b counteracts diffuse large B-cell lymphoma progression via OX40/OX40L-mediated interaction with Th17 cells

Rui Sun[1†], Pei-Pei Zhang[2†], Xiang-Qin Weng[1†], Xiao-Dong Gao[1†], Chuan-Xin Huang[3], Li Wang[1], Xiao-Xia Hu[1], Peng-Peng Xu[1], Lin Cheng[1], Lu Jiang[1], Di Fu[1], Bin Qu[4], Yan Zhao[1], Yan Feng[5], Hong-Jing Dou[2*], Zhong Zheng[1*], Wei-Li Zhao[1*]

[†]Rui Sun, Pei-Pei Zhang, Xiang-Qin Weng and Xiao-Dong Gao contributed equally to this work.

[1]Shanghai Institute of Hematology, State Key Laboratory of Medical Genomics, National Research Center for Translational Medicine at Shanghai, Ruijin Hospital Affiliated to Shanghai Jiao Tong University School of Medicine, Shanghai, China.

[2]State Key Laboratory of Metal Matrix Composites, School of Materials Science and Engineering, National Research Center for Translational Medicine at Shanghai, Shanghai Jiao Tong University, Shanghai, China.

[3]Department of Immunobiology and Microbiology, Shanghai Institute of Immunology, Shanghai Jiao Tong University School of Medicine, Shanghai, China.

[4]Department of Laboratory Medicine, Shanghai RuiJin Hospital, Shanghai Jiao Tong University School of Medicine, Shanghai, China.

[5]State Key Laboratory of Microbial Metabolism, School of Life Sciences and Biotechnology, Shanghai Jiao Tong University, Shanghai, China.

*Corresponding authors: Wei-Li Zhao, Ph.D. Email: zhao.weili@yahoo.com, Zhong Zheng, Ph.D. Email: zheng_zhong89@163.com, Shanghai Institute of Hematology, State Key Laboratory of Medical Genomics, National Research Center for Translational Medicine at Shanghai, Ruijin Hospital Affiliated to Shanghai Jiao Tong University School of Medicine, 197 RuiJin Er Road, Shanghai 200025, China. Tel: 0086-21-64370045, Fax: 0086-21-64743206.

Hong-Jing Dou, Ph.D. Email: hjdou@sjtu.edu.cn, State Key Laboratory of Metal Matrix Composites, School of Materials Science and Engineering, National Research Center for Translational Medicine at Shanghai, Shanghai Jiao Tong University, 800 Dongchuan Road, Shanghai 200240, China. Tel: 0086-21-54748860, Fax: 0086-21-34202749.

This PDF file includes:

Supplementary Table. 1 to Supplementary Table. 2

Supplementary Fig. 1 to Supplementary Fig. 7

Supplementary Table. 1 Univariate analysis for predictors of PFS and OS in DLBCL (n=532)

Characteristics		PFS			OS	
	HR	(95% CI)	P value	HR	(95% CI)	P value
Sex	1.243	0.896-1.725	0.190	1.233	0.835-1.823	0.290
Female						
Male						
Age	1.567	1.135-2.165	0.006	2.471	1.661-3.675	<0.001
> 60 years						
≤ 60 years						
ECOG	2.394	1.688-3.393	<0.001	2.413	1.593-3.657	<0.001
0-1						
2						
Ann Arbor	3.406	2.392-4.851	<0.001	3.349	2.179-5.148	<0.001
I-II						
III-IV						
Extranodal involvement	1.381	0.999-1.909	0.051	1.349	0.914-1.990	0.133
No						
Yes						
LDH	4.060	2.812-5.862	<0.001	3.686	2.369-5.738	<0.001
Normal						
Elevated						
International Prognostic Index (IPI)	3.397	2.441-4.727	<0.001	3.462	2.323-5.162	<0.001
0-2						
3-5						
MiR130b	3.232	2.227-4.690	<0.001	2.642	1.720-4.060	<0.001
Low						
High						

Supplementary Table. 2 Multivariate analysis for predictors of PFS and OS in DLBCL (n=532)

	Variable	HR	(95% CI)	P value
PFS				
	IPI	3.115	2.236-4.339	<0.001
	MiR130b	2.940	2.024-4.272	<0.001
OS				
	IPI	3.147	2.104-4.707	<0.001
	MiR130b	2.302	1.494-3.548	0.008

Sun et al. Supplementary Fig. 1

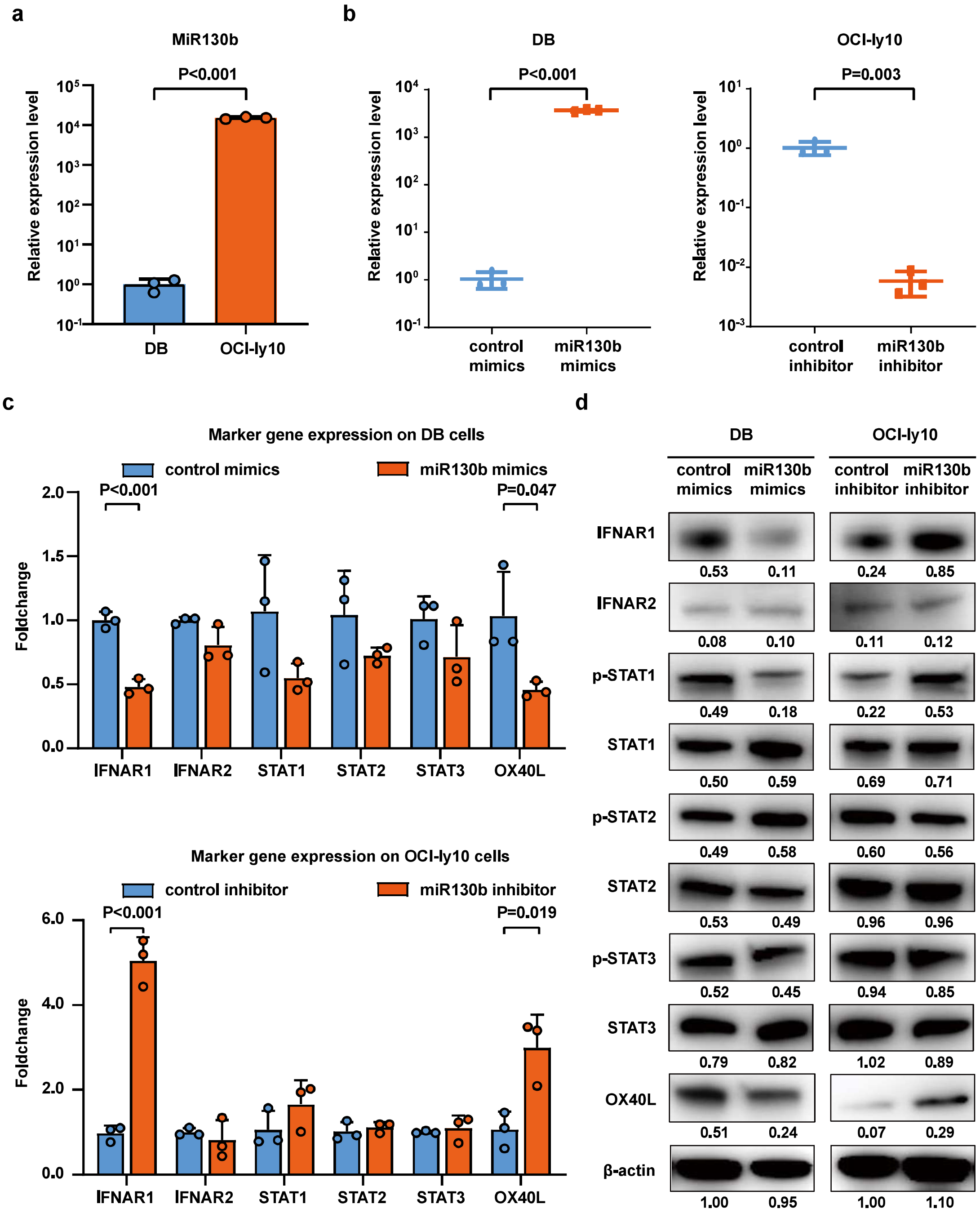

Supplementary Fig. 1 Transfection efficiency of miR130b. a Real-time PCR analysis of miR130b expression in DB cells and OCI-ly10 cells. **b** Real-time PCR analysis of miR130b expression in DB cells transfected with control mimics or miR130b mimics, and OCI-ly10 cells

transfected with control inhibitor or miR130b inhibitor. **c** Real-time PCR analysis of IFNAR1, IFNAR2, STAT1, STAT2, STAT3 and OX40L on DB cells transfected with control mimics, miR130b mimics and OCI-ly10 cells transfected with control inhibitor, miR130b inhibitor. Data are summarized as mean ± SD (n=3). **d** Western blot analysis of IFNAR1, IFNAR2, p-STAT1, STAT1, p-STAT2, STAT2, p-STAT3, STAT3 and OX40L on DB cells transfected with control mimics, miR130b mimics and OCI-ly10 cells transfected with control inhibitor, miR130b inhibitor.

Sun et al. Supplementary Fig. 2

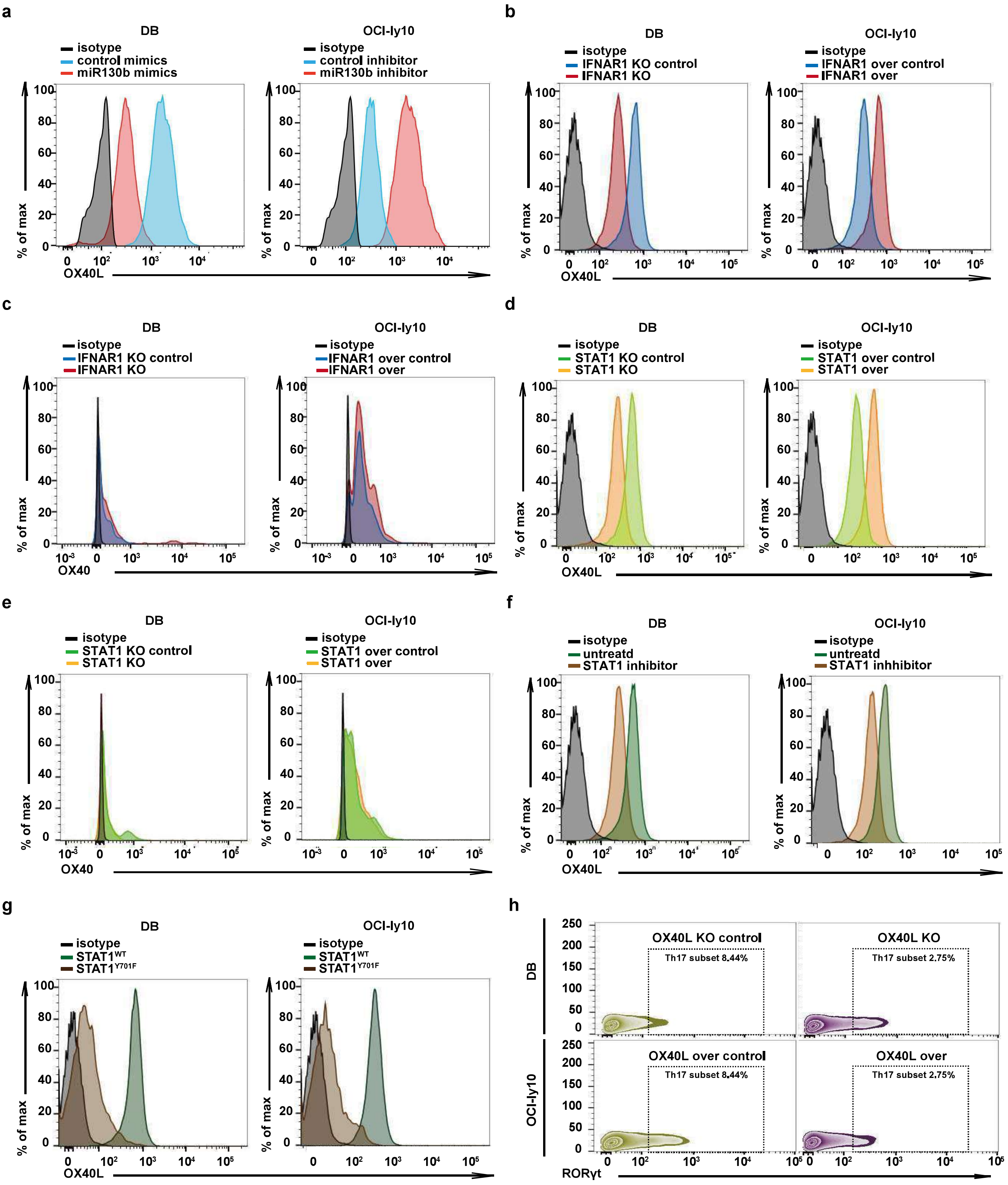

Supplementary Fig. 2 Representative flow cytometry images of miR130b modulated OX40/OX40L-mediated B-lymphoma cell interaction with Th17 cells via IFNAR1/p-STAT1 axis. **a** Representative flow cytometry images of OX40L expression on miR130b mimics

transfected DB co-culture system and miR130b inhibitor transfected OCI-ly10 co-culture system. **b** Representative flow cytometry images of OX40L expression in the IFNAR1-knockdown DB co-culture system and IFNAR1-overexpressing OCI-ly10 co-culture system. **c** Representative flow cytometry images of OX40 expression in the IFNAR1-knockdown DB co-culture system and IFNAR1-overexpressing OCI-ly10 co-culture system. **d** Representative flow cytometry images of OX40L expression in the STAT1-knockdown DB co-culture system and STAT1-overexpressing OCI-ly10 co-culture system. **e** Representative flow cytometry images of OX40 expression in the STAT1-knockdown DB co-culture system and STAT1-overexpressing OCI-ly10 co-culture system. **f** Representative flow cytometry images of OX40L expression in DB cells and OCI-ly10 cells upon treatment with p-STAT1 inhibitor. **g** Representative flow cytometry images of OX40L expression in the $STAT1^{Y701F}$ DB cells and $STAT1^{Y701F}$ OCI-ly10 cells. **h** Representative flow cytometry images of Th17 cell percentage in the OX40L-knockdown DB co-culture system and OX40L-overexpressing OCI-ly10 co-culture system.

Sun et al. Supplementary Fig. 3

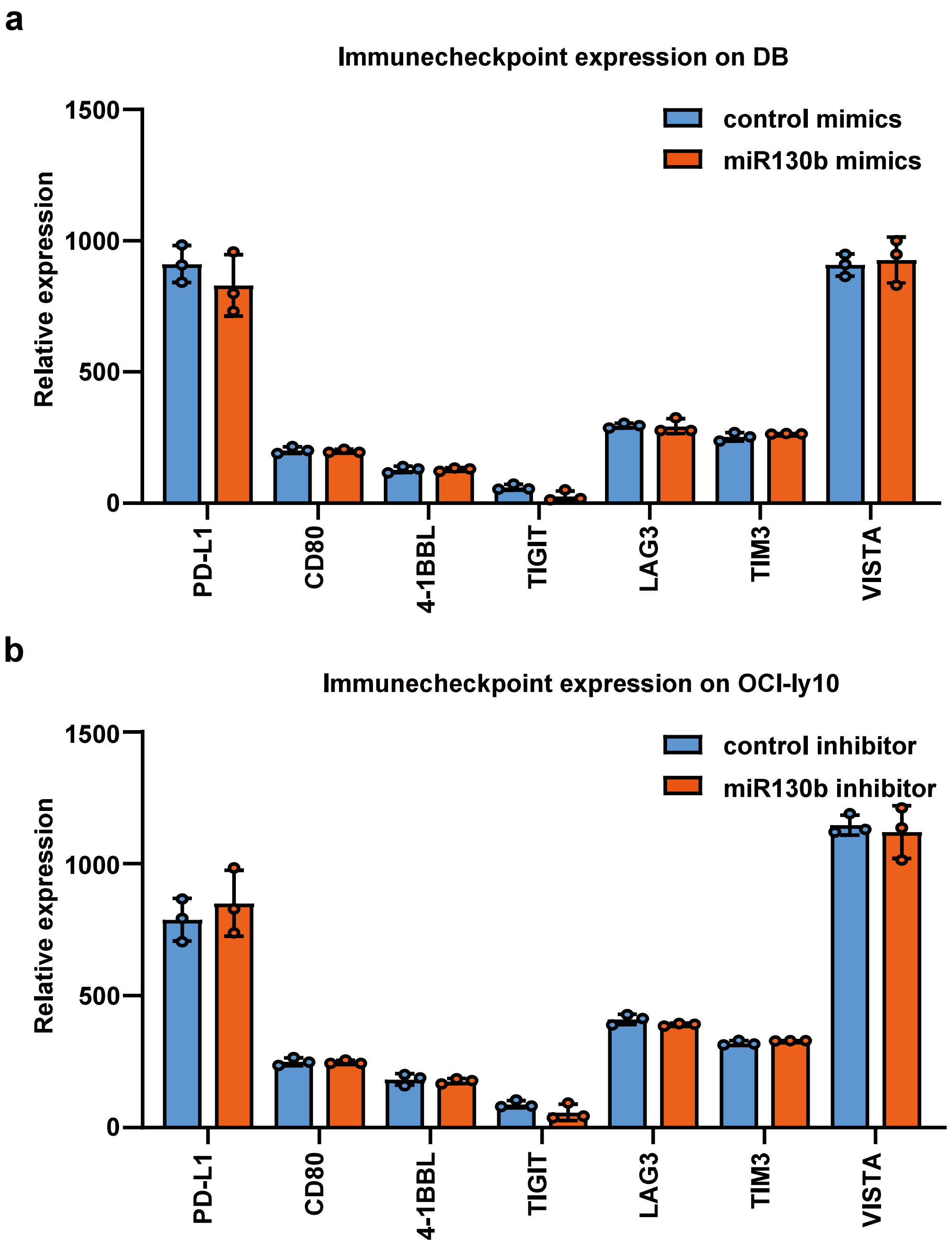

Supplementary Fig. 3 MiR130b regulated immune checkpoint genes. a Flow cytometry analysis of immune checkpoint genes in the control mimics or miR130b mimics transfected DB co-culture system. Data are summarized as mean ± SD (n=3). **b** Flow cytometry analysis of immune checkpoint genes in the control inhibitor or miR130b inhibitor transfeted OCI-ly10 co-culture system. Data are summarized as mean ± SD (n=3).

Sun et al. Supplementary Fig. 4

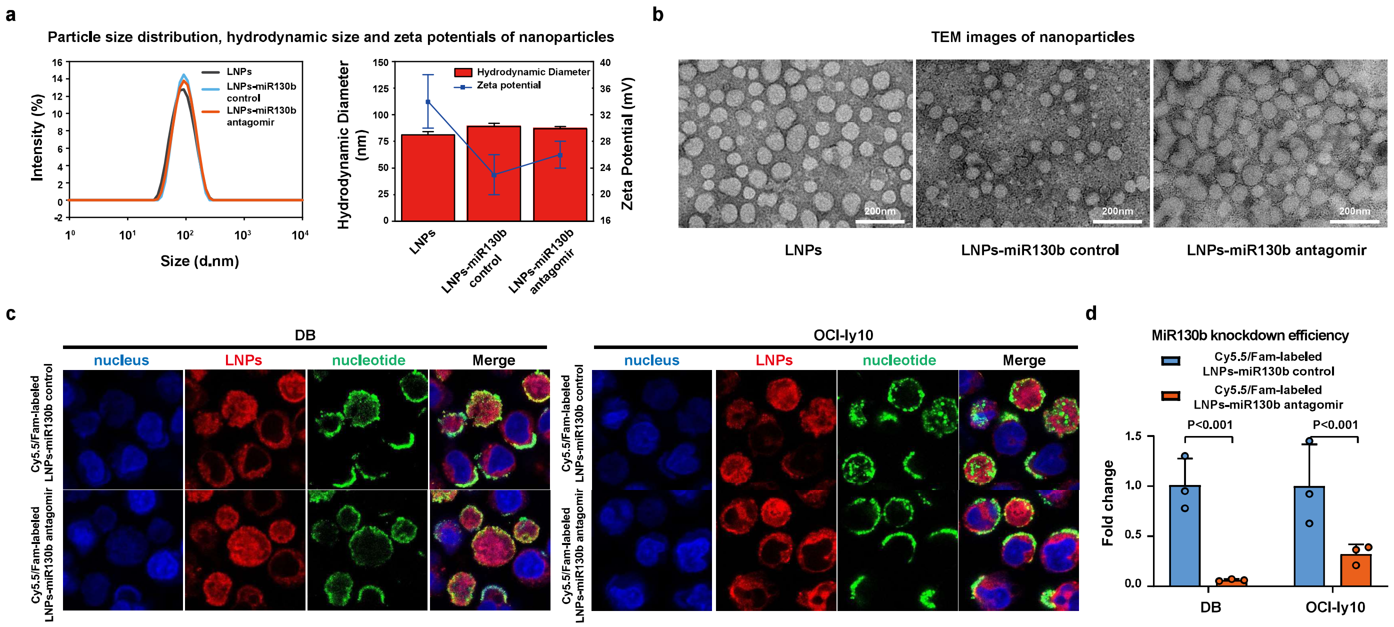

Supplementary Fig. 4 Intracellular distribution and transfection efficiency of Cy5.5/Fam-labeled LNPs-miR130b control and Cy5.5/Fam-labeled LNPs-miR130b. a The particle size distribution, hydrodynamic size and zeta potential of the LNPs, LNPs-miR130b control and LNPs-miR130b antagomir. **b** Transmission electron microscope images of the LNPs, LNPs-miR130b control and LNPs-miR130b antagomir (negatively stained by phosphotungstic acid). **c** Immunofluorescence assay of LNPs and nucleotide in DB cells (left panel) and OCI-ly10 cells (right panel) transfected with Cy5.5/Fam-labeled LNPs-miR130b control or Cy5.5/Fam-labeled LNPs-miR130b antagomir. **d** Real-time PCR analysis of miR130b expression in DB cells transfected with Cy5.5/Fam-labeled LNPs-miR130b control or Cy5.5/Fam-labeled LNPs-miR130b antagomir and OCI-ly10 cells transfected with Cy5.5/Fam-labeled LNPs-miR130b control or Cy5.5/Fam-labeled LNPs-miR130b antagomir. Data are summarized as mean ± SD (n=3).

Sun et al. Supplementary Fig. 5

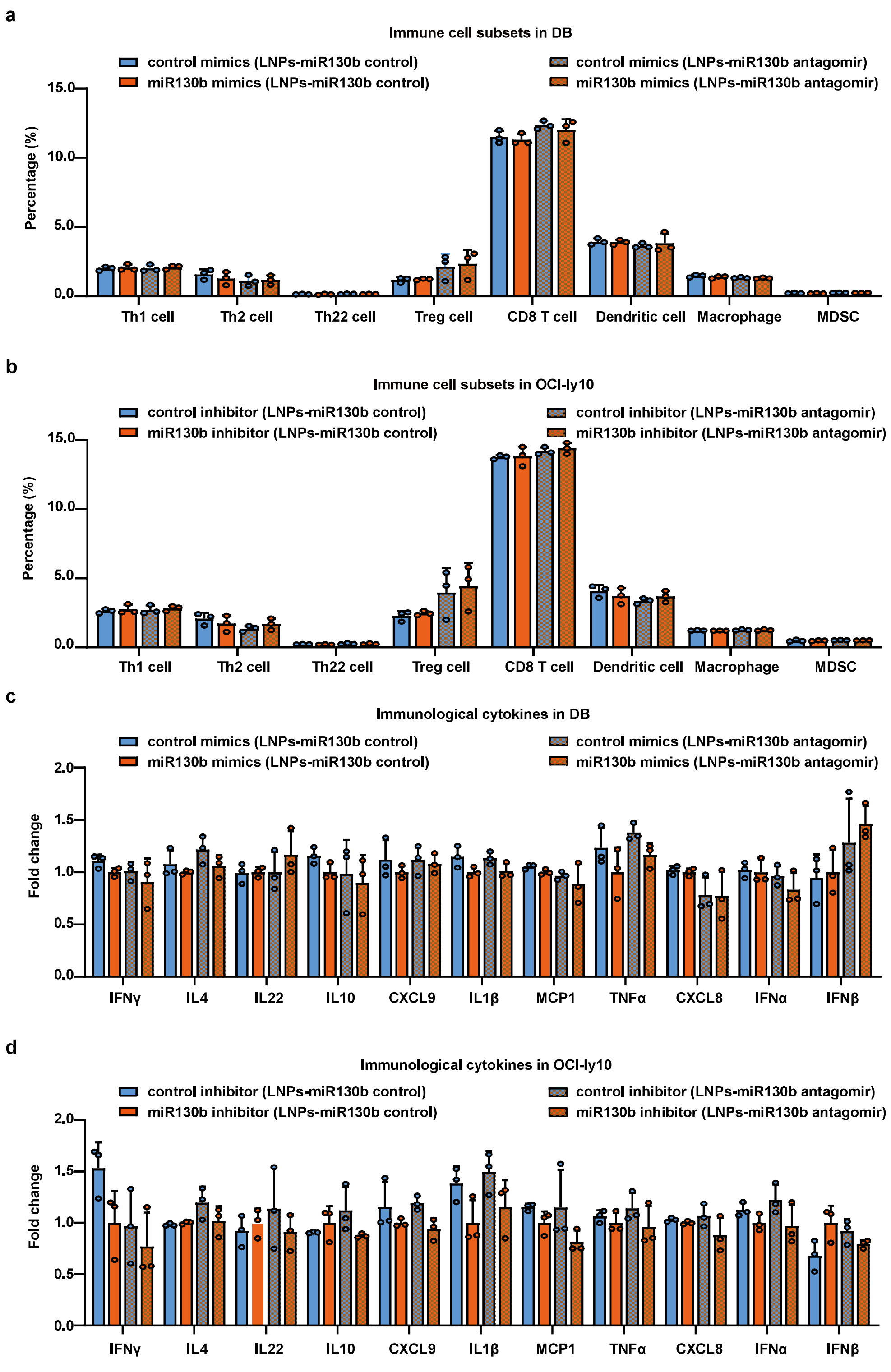

Supplementary Fig. 5 MiR130b regulated immune cell accumulation and cytokine secretion

a Flow cytometry analysis of immune cell subsets in the control mimics or miR130b mimics transfected DB co-culture system upon treatment with LNPs-miR130b control or LNPs-miR130b

antagomir. Data are summarized as mean ± SD (n=3). **b** Flow cytometry analysis of immune cell subsets in the control inhibitor or miR130b inhibitor transfected OCI-ly10 co-culture system upon treatment with LNPs-miR130b control or LNPs-miR130b antagomir. Data are summarized as mean ± SD (n=3). **c** ELISA analysis of related immunological cytokines in the control mimics or miR130b mimics transfected DB co-culture system upon treatment with LNPs-miR130b control or LNPs-miR130b antagomir. Data are summarized as mean ± SD (n=3). **d** ELISA analysis of related immunological cytokines in the control inhibitor or miR130b inhibitor transfected OCI-ly10 co-culture system upon treatment with LNPs-miR130b control and LNPs-miR130b antagomir. Data are summarized as mean ± SD (n=3).

Sun et al. Supplementary Fig. 6

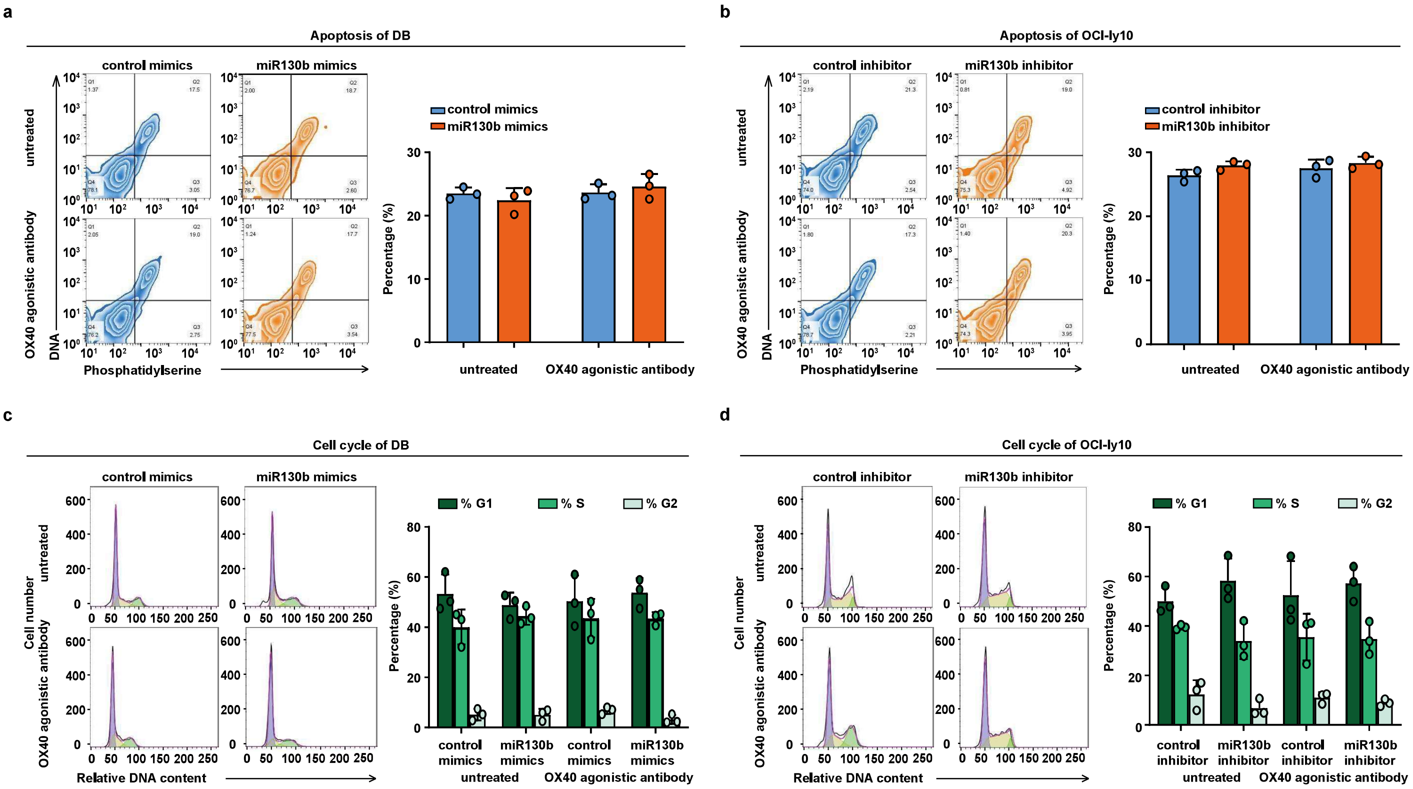

Supplementary Fig. 6 MiR130b mediated tumor apoptosis and cell cycle. a Flow cytometry analysis of DB cell apoptosis in the control mimics or miR130b mimics transfected DB co-culture system upon treatment with or without OX40 agonistic antibody. **b** Flow cytometry analysis of OCI-ly10 cell apoptosis in the control inhibitor or miR130b inhibitor transfected OCI-ly10 co-culture system upon treatment with or without OX40 agonistic antibody. **c** Flow cytometry analysis of DB cell cycle in the control mimics or miR130b mimics transfected DB co-culture system upon treatment with or without OX40 agonistic antibody. **d** Flow cytometry analysis of OCI-ly10 cell cycle in the control inhibitor or miR130b inhibitor transfected OCI-ly10 co-culture system upon treatment with or without OX40 agonistic antibody.

Sun et al. Supplementary Fig. 7

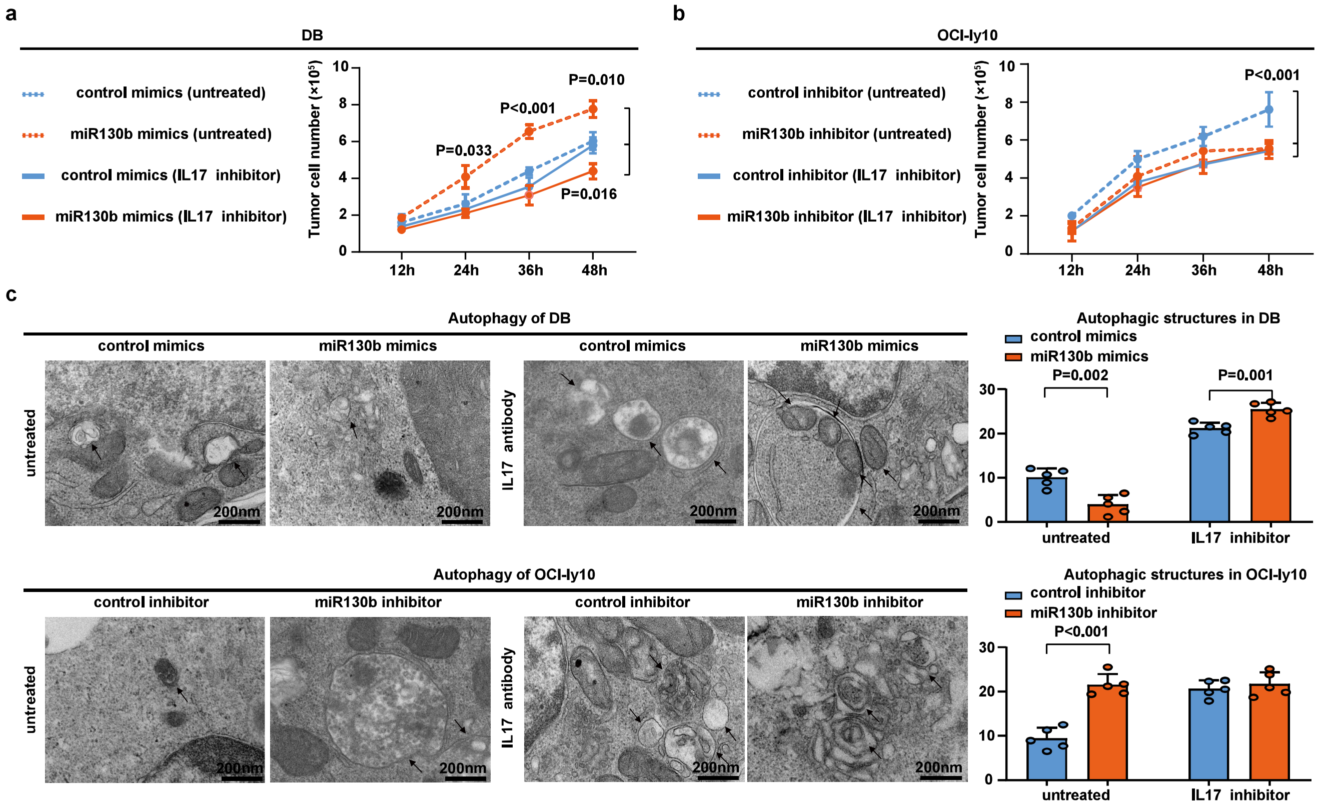

Supplementary Fig. 7 IL17 inhibitor mediated tumor autophagy. a Cell growth in the control mimics or miR130b mimics transfected DB co-culture system upon treatment with or without IL17 inhibitor. MTT assay was adopted to measure cell viability. Data are summarized as mean ± SD (n=3). **b** Cell growth in the control inhibitor or miR130b inhibitor transfected OCI-ly10 co-culture system upon treatment with or without IL17 inhibitor. MTT assay was adopted to measure cell viability. Data are summarized as mean ± SD (n=3). **c** Transmission electron microscope showed typical autophagosomes in the control mimics or miR130b mimics transfected DB co-culture system and control inhibitor or miR130b inhibitor transfected OCI-ly10 co-culture system upon treatment with or without IL17 inhibitor. The cells were counted from five visions selected at random and subjected for statistical analysis. Data are summarized as mean ± SD (n=5).
